# Diversity of Chromanol and Chromenol Structures and Functions: An Emerging Class of Anti-Inflammatory and Anti-Carcinogenic Agents

**DOI:** 10.3389/fphar.2020.00362

**Published:** 2020-04-21

**Authors:** Maria Wallert, Stefan Kluge, Martin Schubert, Andreas Koeberle, Oliver Werz, Marc Birringer, Stefan Lorkowski

**Affiliations:** ^1^ Department of Biochemistry and Physiology of Nutrition, Institute of Nutritional Sciences, Friedrich Schiller University Jena, Jena, Germany; ^2^ Department of Pharmaceutical/Medicinal Chemistry, Institute of Pharmacy, Friedrich Schiller University Jena, Jena, Germany; ^3^ Michael Popp Research Institute, University of Innsbruck, Innsbruck, Austria; ^4^ Department of Nutrition, Food and Consumer Sciences, University of Applied Sciences Fulda, Fulda, Germany; ^5^ Regionales Innovationszentrum Gesundheit und Lebensqualität (RIGL), Fulda, Germany; ^6^ Competence Center for Nutrition and Cardiovascular Health (nutriCARD) Halle-Jena-Leipzig, Jena, Germany

**Keywords:** chromanols, chromenols, inflammation, cancer, molecular targets

## Abstract

Natural chromanols and chromenols comprise a family of molecules with enormous structural diversity and biological activities of pharmacological interest. A recently published systematic review described more than 230 structures that are derived from a chromanol ortpd chromenol core. For many of these compounds structure-activity relationships have been described with mostly anti-inflammatory as well as anti-carcinogenic activities. To extend the knowledge on the biological activity and the therapeutic potential of these promising class of natural compounds, we here present a report on selected chromanols and chromenols based on the availability of data on signaling pathways involved in inflammation, apoptosis, cell proliferation, and carcinogenesis. The chromanol and chromenol derivatives seem to bind or to interfere with several molecular targets and pathways, including 5-lipoxygenase, nuclear receptors, and the nuclear-factor “kappa-light-chain-enhancer” of activated B-cells (NFκB) pathway. Interestingly, available data suggest that the chromanols and chromenols are promiscuitively acting molecules that inhibit enzyme activities, bind to cellular receptors, and modulate mitochondrial function as well as gene expression. It is also noteworthy that the molecular modes of actions by which the chromanols and chromenols exert their effects strongly depend on the concentrations of the compounds. Thereby, low- and high-affinity molecular targets can be classified. This review summarizes the available knowledge on the biological activity of selected chromanols and chromenols which may represent interesting lead structures for the development of therapeutic anti-inflammatory and chemopreventive approaches.

## Introduction

**Figure 1:**
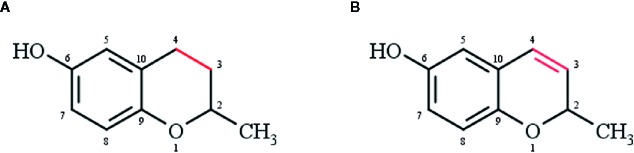
**(A)** Chromanol (2-methyl-3,4-dihydro-2*H*-chromen-6-ol) and **(B)** chromenol (2-methyl-2*H*-chromen-6-ol) core structure.

Chromanols and chromenols are collective terms for about 230 structures derived from photosynthetic organisms like plants, algae, cyanobacteria, fungi, corals, sponges, and tunicates (Birringer et al., 2018). Both compound classes are formed by a cyclization of substituted 1,4-benzoquinones. While 6-hydroxy-chromanols are derived from a 2-methyl-3,4-dihydro-2*H*-chromen-6-ol structure, 6-hydroxy-chromenols are derived from 2-methyl-2*H*-chromen-6-ol (**Figure 1**). The respective bicyclic core structure is associated to a side-chain with varying chain length and modifications, resulting in a great diversity of chromanol and chromenol derivates (Birringer et al., 2018). In a systematic review, Birringer and coworkers were the first implying the great potential of these structures by providing a comprehensive overview of the structural diversity and chemical transformation of all 230 chromanols and chromenols known at that time together with their natural source. The aim of the comprehensive review was rather the detailed description of the complexity of this group of compounds than an outline of their biological activity. Based on this systematic review, the intention of our review was to more selectively describe the effects of this class of natural products on signaling pathways involved in inflammation, apoptosis, cell proliferation, and carcinogenesis, and the underlying molecular modes of action for selected chromanols and chromenols. Our review therefore represents a useful and relevant addition to the work of Birringer et al., focusing on the evaluation of selected compounds with known biological activity as possible lead structures for putative therapeutic approaches. Based on the mentioned inclusion criteria, we here focus on tocopherol (TOH) and tocotrienol (T3) structures, sargachromanols, amplexichromanols, and sargachromenols, which show structure-activity relationships with mostly anti-inflammatory as well as anti-carcinogenic activities.

Tocopherols and T3s differ in the saturation of the side-chain and form in its entirety the group of vitamin E. Based on the methylation pattern of the chromanol ring system α-, β-, γ-, δ-forms of TOHs and T3s can be distinguished. Oxidative modifications of the terminal side-chain increase anti-inflammatory activities. Therefore, hepatic metabolites of vitamin E are supposed to have important physiological activities and will also be included in this review. Sargachromanols (SCA), sargachromenols (SCE), and amplexichromanols (AC) have a tocotrienol-derived backbone implying similar biological activities. Our review focuses in more detail on the current knowledge about the biological activity as well as on potential regulatory pathways and molecular targets of chromanols and chromenols.

## Chromanol and Chromenol Structures

### Chromanols

#### Tocopherols and Tocotrienols

**Figure 2:**
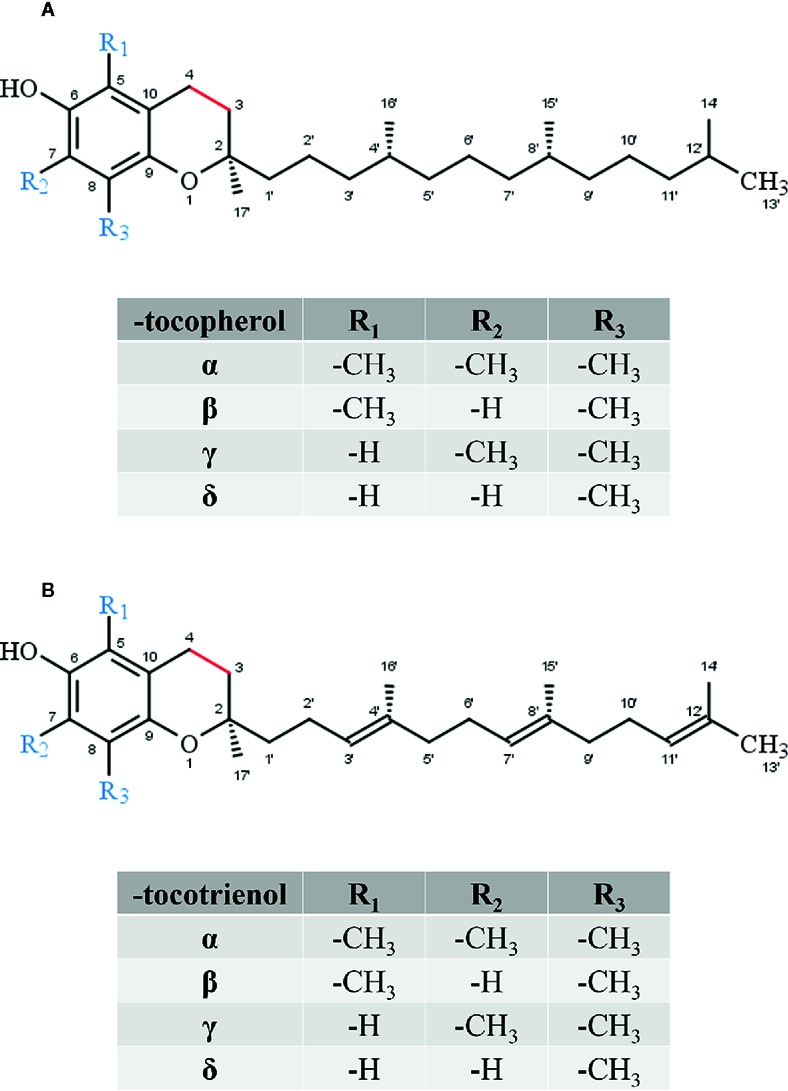
Core structure of **(A)** tocopherol and **(B)** tocotrienol forms.

Vitamin E, more precisely *RRR*-α-tocopherol, has been identified in 1922 as a vital factor for fertility in rats (Evans and Bishop, 1922). Vitamin E does naturally occur in various plant-derived foods, such as oils, nuts, germs, seeds as well as vegetables and, in lower amounts, fruits. Thus, vitamin E represents the most widely distributed and abundant chromanol in nature. The term vitamin E comprises different lipophilic molecules that consist of the chromanol ring structure with a covalently bound phytyl-like side-chain. Depending on the saturation of the C-16′ side-chain, these molecules are classified as TOH, T3s (**Figure 2**), and vitamin E related structures named tocomonoenols and marine-derived TOHs. Tocopherols are characterized by a saturated phytyl side-chain whereas tocomonoenols, marine-derived TOHs and T3 are unsaturated at either the terminal isoprene unit or have three double bonds within the side-chain (Fujisawa et al., 2010; Kruk et al., 2011). Further, the methylation pattern of the chromanol ring determines the classification as α-, β-, γ-, and δ-forms of TOHs and T3s. Although several similar molecules form the group of vitamin E, only α-TOH seems to have vitamin property in animals and humans. For instance, in rats α-TOH preserves fertility, whereas in humans the deficiency disease *ataxia with vitamin E deficiency* (AVED) is prevented by α-TOH supplementation (Azzi, 2019).

For a long time, the health-promoting effects of vitamin E were only attributed to its antioxidant properties, but more recent studies revealed additional non-antioxidant functions of vitamin E. It is evident that vitamin E modulates gene expression and enzyme activities and also interferes with signaling cascades (Brigelius-Flohé, 2009; Zingg, 2019). Examples for these regulatory effects are the suppression of inflammatory mediators, reactive oxygen species (ROS) and adhesion molecules, the induction of scavenger receptors as well as the activation of nuclear factor kappa-light-chain-enhancer of activated B cells (NF-кB) (reviewed in Glauert, 2007; Rimbach et al., 2010; Wallert et al., 2014b; Zingg, 2019).

**Figure 3:**
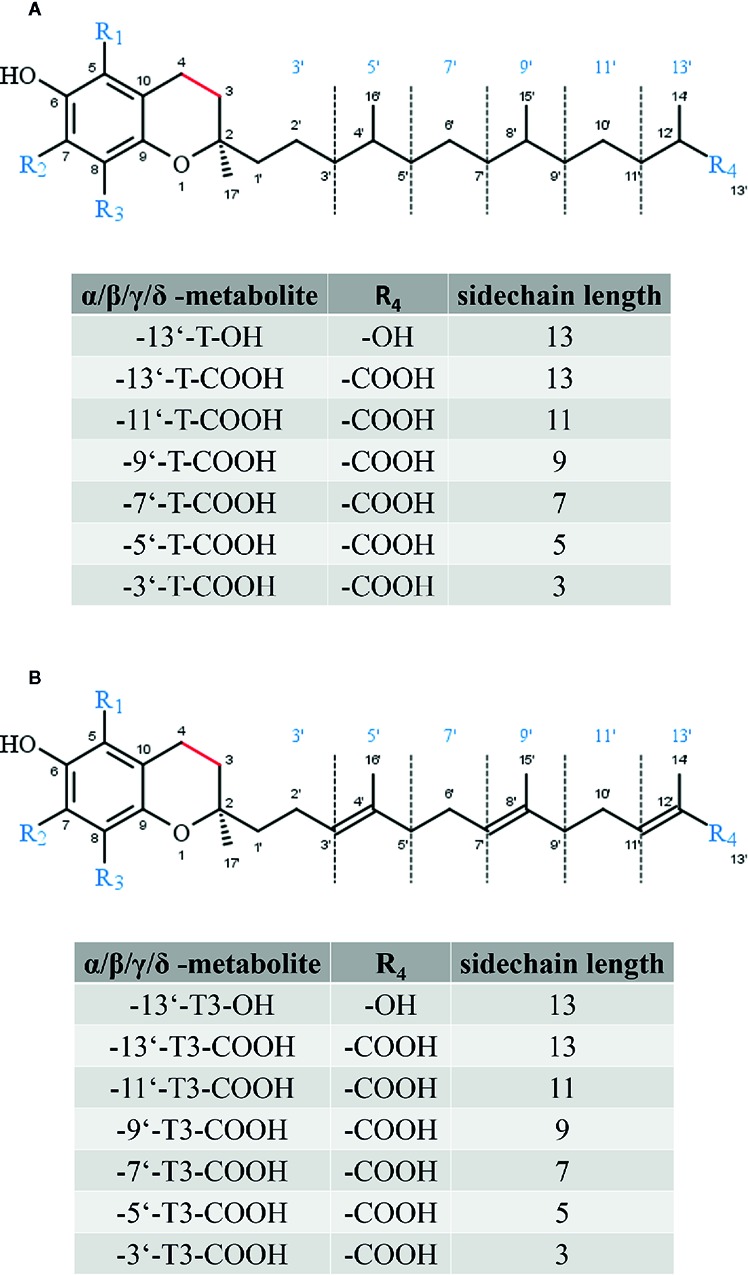
Core structure of **(A)** tocopherol and **(B)** tocotrienol metabolites. The substitutions R1 to R3 are given in **Figure 2** **(A)** and **(B)**.

All forms of vitamin E undergo metabolic degradation in the liver. Although the detailed mechanisms remain poorly understood, the principles of the degradation of vitamin E to vitamer-specific physiological metabolites with intact chromanol ring (the nomenclature as α-, β-, γ- and δ-metabolites is used as described for the metabolic precursors in order to distinguish the different forms of vitamin E metabolites) is widely accepted (**Figure 3**). Thus, enzymatic modifications are restricted to the side-chain (extensively reviewed in (Kluge et al., 2016; Schmölz et al., 2016)). α-Tocopherol is the main form of vitamin E in the human body due to its higher binding affinity to the α-tocopherol transfer protein (Hosomi et al., 1997). Thus, we will focus on the metabolic conversion of α-TOH in the following. Nevertheless, it should be noted that all forms of vitamin E (TOHs as well as T3s) follow the same metabolic route. However, due to the unsaturated side-chain, the degradation of T3s requires further enzymes such as 2,4 dienoyl-coenzyme A (CoA) reductase and 3,2-enoyl-CoA isomerase, which are also involved in the metabolism of unsaturated fatty acids (Birringer et al., 2002). The initial step of α-TOH modification *via* ω-hydroxylation in the endoplasmic reticulum leads to the formation of the long-chain metabolite (LCM) α-13′-hydroxychromanol (α-T-13′-OH). It is supposed that this hydroxylation is catalyzed by cytochrome P450 (CYP)4F2 and CYP3A4 (Parker et al., 2000; Sontag and Parker, 2002). After its transfer from the endoplasmic reticulum to the peroxisome, α-T-13′-OH is converted to α-13′-carboxychromanol (α-T-13′-COOH) *via* ω-oxidation, likely *via* a two-step mechanism involving alcohol and aldehyde dehydrogenases. α-T-13′-OH and α-T-13′-COOH have been found in human serum (Wallert et al., 2014a; Ciffolilli et al., 2015; Giusepponi et al., 2017), supporting the idea of a more complex physiologic role of vitamin E with physiological relevance of its metabolites for various processes. In healthy humans α-TOH is the most abundant form of vitamin E, occurring in concentrations of about 20–30 µM in serum (Péter et al., 2015). However, supplementation of α-TOH increases α-TOH serum concentration in humans up to 90 µM (Dieber-Rotheneder et al., 1991). Following supplementation, the hepatic metabolism is enhanced to protect the liver from excessive accumulation of α-TOH. Consequently, metabolites of vitamin E are formed and accumulate in turn in human serum. The LCMs α-T-13′-OH and α-T-13′-COOH were found in concentrations of 1–7 nM and 1–10 nM at baseline, respectively, whereas supplementation of α-TOH increased serum concentrations of the LCMs up to 12–32 nM and 3–55 nM, respectively (Wallert et al., 2014a; Ciffolilli et al., 2015; Giusepponi et al., 2017). Recent studies showed that the active metabolites of vitamin E exert effects on lipid metabolism, apoptosis, proliferation, and inflammatory processes as well as xenobiotic metabolism (Wallert et al., 2014a; Jang et al., 2016; Podszun et al., 2017; Schmölz et al., 2017). Finally, α-T-13′-COOH is excreted *via* bile and feces or is further degraded *via* several rounds of oxidation to the hydrophilic short-chain metabolite α-carboxyethyl-hydroxychromanol (CEHC), which is largely excreted *via* urine (Zhao et al., 2010; Johnson et al., 2012; Jiang, 2014). Another characteristic of the hepatic degradation of vitamin E is that the metabolites are chemically modified. In particular, the LCMs and the short-chain metabolites (SCMs) have been found as sulfated or glucuronidated conjugates in different biological matrices (Galli et al., 2002; Wallert et al., 2014a). Freiser and Jiang (2009) reported that more than 75% of γ-CEHC in the plasma of γ-T3-supplemented rats occurred in conjugated form. Further, also the LCMs, especially 13′-COOH and 11′-COOH metabolites were found as conjugates. Conjugation (sulfation or glucoronidation) seems to occur in the liver in parallel to the β-oxidation of the side-chain of vitamin E (Freiser and Jiang, 2009).

Beside the mentioned LCMs, intermediate-chain metabolites (ICMs) and SCMs that are formed *via* hepatic degradation of the different vitamin E forms, and vitamin E is also the precursor of quinones, representing another class of vitamin E-derived metabolites that exhibit antioxidant activity. Vitamin E quinones, in particular α-TOH-derived quinones, are formed as byproducts of α-TOH oxidation during peroxidation reactions in *in vitro* systems (Liebler et al., 1990; Infante, 1999). In addition, these metabolites can also be synthesized by photosynthetic organisms (Liebler et al., 1990). Although the knowledge on this group of tocopherol-derived metabolites is sparse, α-TOH quinone has been described as an essential enzymatic cofactor for fatty acid desaturase (Liebler et al., 1990).

The natural compound δ-T3-13′-COOH, also known as δ-garcinoic acid or δ-tocotrienolic acid, shares structural similarity with the δ-T-LCM δ-T-13′-COOH, the second LCM originating from the hepatic metabolism of δ-TOH. As described previously, hepatic metabolism of tocotrienols follows that of tocopherols. Consequently, δ-T3-13′-COOH is formed during the degradation of δ-T3. Since the concentration of δ-T3 in human plasma is below 1% compared to α-TOH, the physiological relevance of δ-T3-13′-COOH in humans is likely low. So far, the detection of δ-T3-13′-COOH in human blood is still pending. However, local accumulation of δ-T3-13′-COOH in cells or tissues cannot be excluded. δ-T3-13′-COOH can be obtained in relatively high amounts and purity from the seeds of *Garcinia kola E. Heckel* (Bartolini et al., 2019; Wallert et al., 2019), a plant that is used in traditional African ethnomedicine (extensively reviewed in Kluge et al., 2016). This compound can be used as precursor for the semi-synthesis of α- and δ-LCMs (including α-T-13′-OH, α-T-13′-COOH, δ-T-13′-OH, and δ-T-13′-COOH) for experimental use *in vitro* and in mice and is therefore important for vitamin E metabolite research (Maloney and Hecht, 2005; Birringer et al., 2010). Further, δ-T3-13′-COOH also appeared to be a potent anti-inflammatory (Wallert et al., 2019) and anti-proliferative agent (Mazzini et al., 2009) and has been shown to act as an inhibitor of DNA polymerase β (Maloney and Hecht, 2005), indicating that δ-T3-13′-COOH may disturb base excision repair in tumor cells. A recent preprint of Bartolini et al. described δ-T3-13′-COOH as a potent agonist of PXR, which is known to be involved in inflammatory processes (Bartolini et al., 2019).

#### Sargachromanols

**Figure 4:**
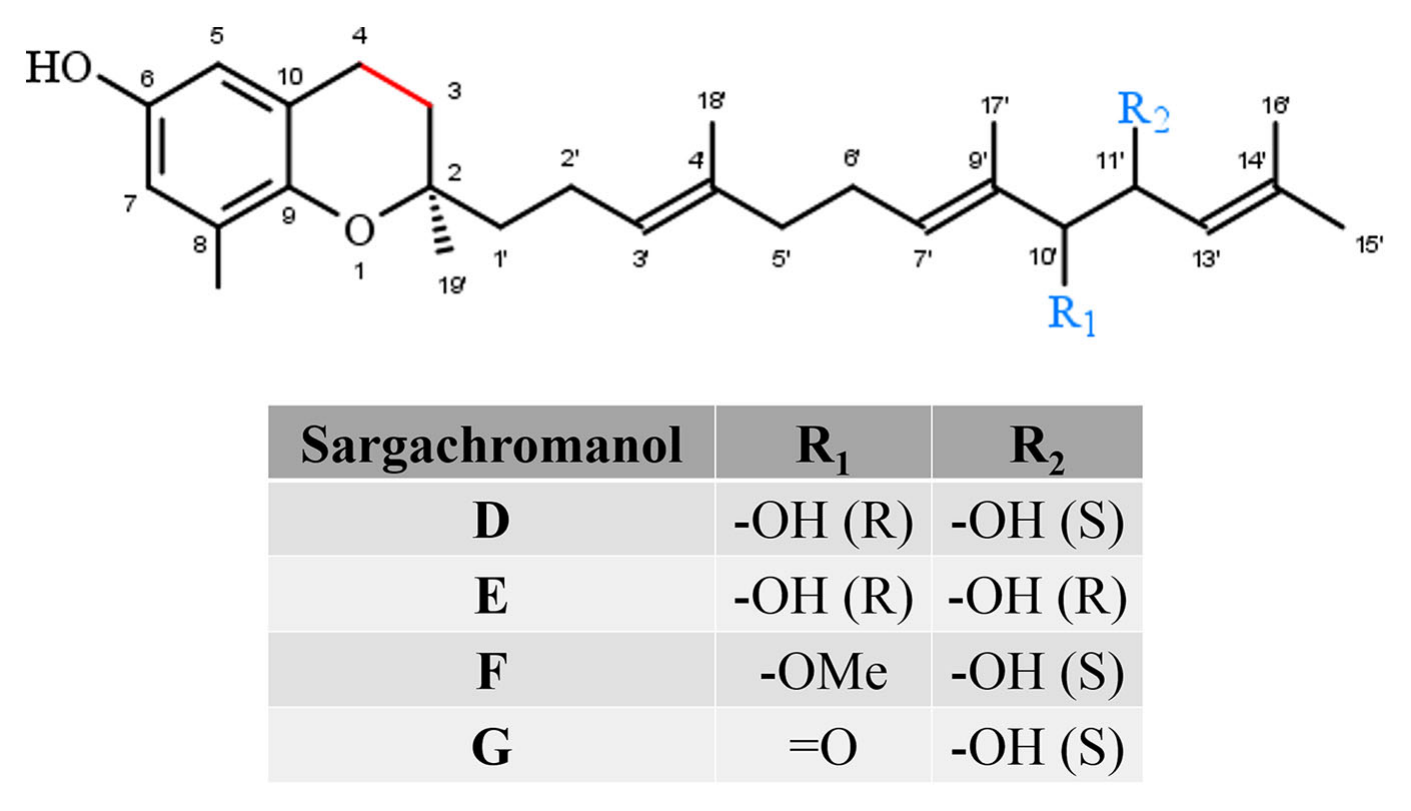
Core structure of sargachromanol forms.

Sargachromanols (SCA) comprise a group of chromanols that occur in the brown algae family *Sargassaceae* (**Figure 4**). Their high structural diversity results from various side-chain modifications, leading to their classification from SCA-A to SCA-S. The entirety of sargachromanols has been isolated from *Sargassum siliquastrum* and has been classified *via* two-dimensional nuclear magnetic resonance experiments (Jang et al., 2005; Im Lee and Seo, 2011). The extensive analysis revealed detailed structural differences between the sargachromanols. For example SCA-C contains a 9′-hydroxyl group with *R*-configuration in the side-chain, while SCA-F has a methoxy group at C-9′ and a hydroxyl group with *R*-configuration at C-10′ (extensively reviewed in Birringer et al., 2018). SCAs have been reported to exhibit various biological activities, including anti-oxidative (Lim et al., 2019) (SCA-G), anti-osteoclastogenic (Yoon et al., 2012b; Yoon et al., 2013) (SCA-G), anti-inflammatory (Yoon et al., 2012a; Lee et al., 2013; Heo et al., 2014) (SCA-G and SCA-D), as well as anti-diabetic (Pak et al., 2015) (SCA-I) ones. To the best of our knowledge, metabolism of sargachromanols in humans or animals has not been investigated.

#### Amplexichromanols

**Figure 5:**
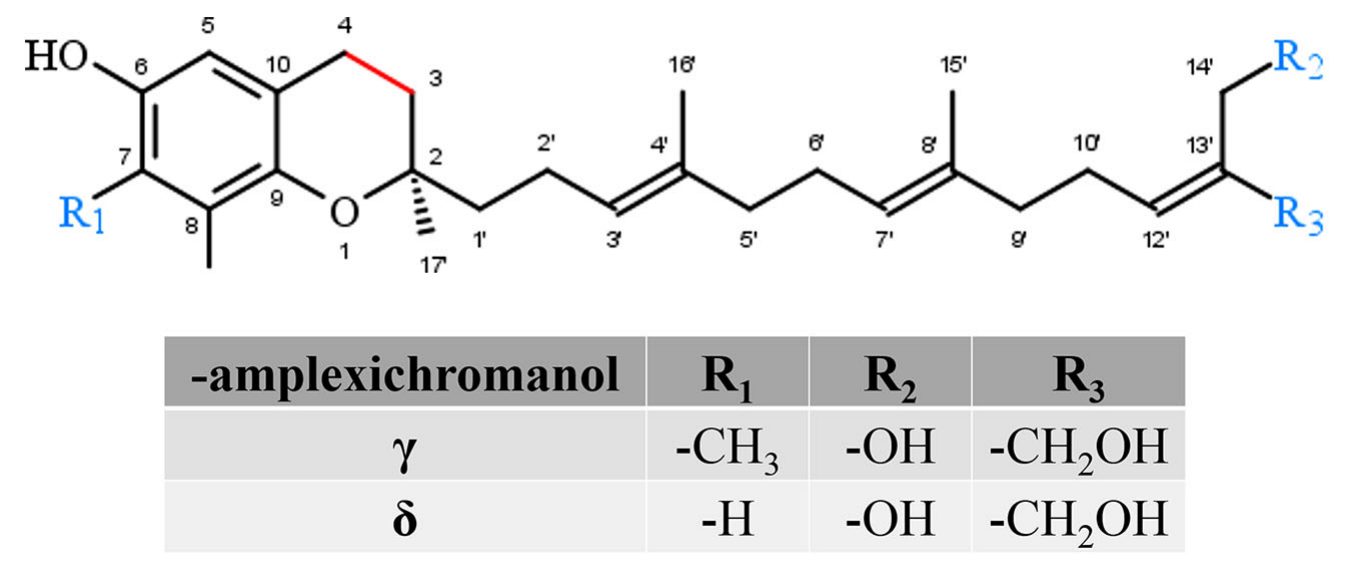
Core structure of amplexichromanol forms.

Amplexichromanols represent a small group of hydroxylated T3 derivatives found in different parts of *Garcinia* plants. For instance, lipophilic extracts from the bark of *Garcinia amplexicaulis* were used to isolate γ-AC and δ-AC (**Figure 5**). The chemical structure of γ-AC and δ-AC are similar to γ-T3 and δ-T3, respectively, but carry two additional hydroxyl groups at C-13′ and C-14′. In an initial *in vitro* experiment, δ-AC reduced vascular endothelial growth factor induced cell proliferation in low nanomolar concentrations, while γ-AC had no effect. This observation probably indicates distinct efficiencies for the different amplexichromanols (Lavaud et al., 2013). However, further experiments revealed strong anti-oxidative potential for both compounds (Lavaud et al., 2015), but nothing is known about the metabolization, systemic distribution, tissue accumulation, or excretion of amplexichromanols so far.

### Chromenols

**Figure 6:**
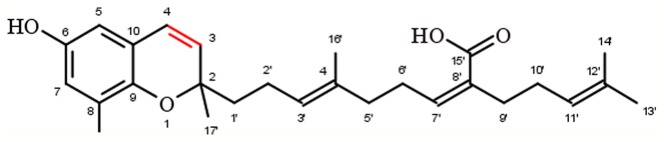
Molecular structure of δ-sargachromenol.

Chromenols consist of a 2-methyl-2*H*-chromen-6-ol core that is associated with a side-chain with varying chain length and varying chemical modifications, leading to high structural diversity. The multitude of these compounds can be obtained from photosynthetic organisms like plants, algae, cyanobacteria, fungi, corals, sponges, and tunicates (Birringer et al., 2018). As the current knowledge on the biological functions of chromenol structures is sparse, this review will exemplarily focus on the most studied sargachromenols (**Figure 6**). Similar to their chromanol counterparts, sargachromenols were named after the brown algae species *Sargassum serratifolium*, from which they have been isolated first (Kusumi et al., 1979). Just like sargachromanols, sargachromenols comprise a molecule class of high structural diversity due to different side-chain modifications. In the first systematic review on the field of chromanols and chromenols, Birringer and coworkers described 15 sargachromenols, 13 compounds with marine origin (brown algae) and two with marine and plant origin (Birringer et al., 2018). As an example, δ-SCE, a structure consisting of a δ-chromenol ring system with an unsaturated side-chain containing a carboxy group at C-15′, is widely distributed in algae of the *Sargassaceae* family but can also be obtained from plants like *Iryanthera juruensis*. Another interesting sargachromenol is dehydro-δ-T3, or Sargol, which is supposed to serve as a biosynthetic precursor for most of the sargachromenols and is occurring in brown algae (Birringer et al., 2018). Brown algae from the *Sargassaceae* family have been used in traditional Asian medicine as well as in health promoting diets, revealing a variety of biological functions (Kim et al., 2014). For example, ethanolic extracts from the *Sargassaceae* species *Myagropsis myagroides*, an alga that grows at the coast of East Asia, revealed potent anti-inflammatory activity. After HPLC-based separation, sargachromenols (mostly δ-SCE) have been identified as the most potent anti-inflammatory compounds within these extracts, based on their inhibitory effect on nitric oxide (NO) production in lipopolysaccharide (LPS)-treated immortalized murine microglial BV-2 cells (Kim et al., 2014). Beside their anti-inflammatory activity, anti-carcinogenic (Hur et al., 2008), anti-photoaging (Kim et al., 2012), and anti-cholinesterase activities (Choi et al., 2007) have been described for SCEs. Further, sargachromenols isolated from *Sargassum macrocarpum* mediate nerve-growth-factor-driven neuronal growth in pheochromocytoma of rat adrenal medulla derived PC12D cells (Tsang et al., 2005).

## Biological Activity of Natural Chromanols and Chromenols

**Figure 7:**
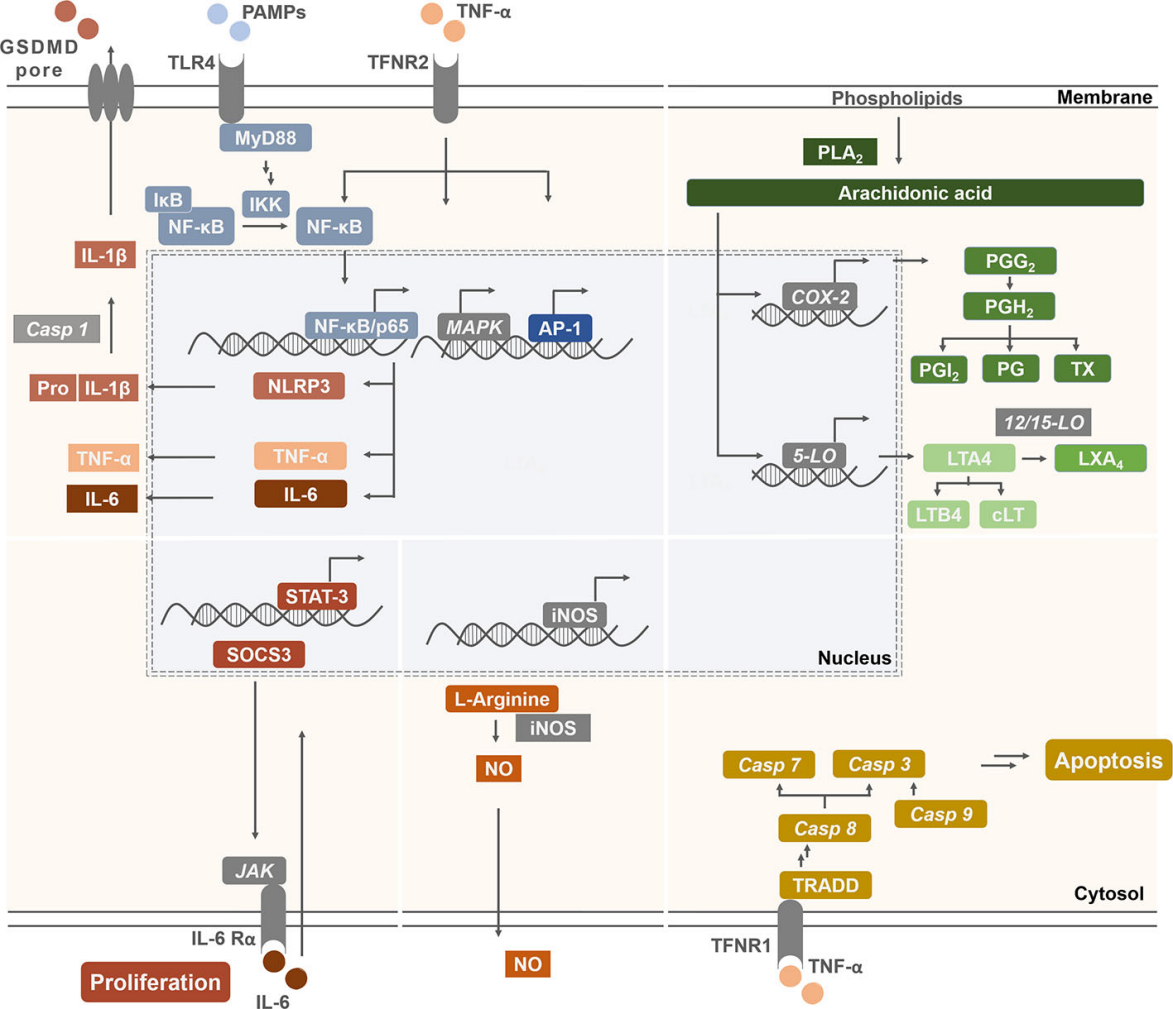
Schematic illustration of signaling targets, pathways, and molecules involved in inflammatory response and cancer progression. Pathways were chosen due to known interactions with the compounds of interest. Inflammatory signaling molecules are interleukins (IL), tumor necrosis factor-α (TNF-α), nitric oxide (NO), prostaglandins (PG), prostacyclin (PGI_2_), tromboxanes (TX), leukotriens (LT), and lipoxins (LX). Their expression, synthesis, or release depends, among others, on the activation of NF-κB, NLR family pyrin domain containing 3 (NLRP3) inflammasome, inducible nitric oxide synthase (iNOS), cyclooxygenase (COX)-2, and lipoxygenases (LO). In addition, membrane receptors, such as cytokine or epidermal growth factor receptors regulate the activation of nuclear receptor signal transducer and activator of transcription (STAT) 3 as well as extrinsic or intrinsic pathways which trigger the activation of caspases (Casp). These mediators are therefore important factors for modulating the balance between cell proliferation and apoptosis, which is essential to prevent carcinogenesis.

Based on published data, we have chosen signaling pathways that are central for inflammation, apoptosis, cell proliferation, and carcinogenesis (**Figure 7**). Respective effects of tocopherol-derived (T) and tocotrienol-derived (T3) chromanol and chromenol structures on nuclear receptors and target enzymes were screened and are discussed in the following.

### Inflammation

Inflammation is essential for wound healing as well as defense and clearance of pathogens (Kunnumakkara et al., 2018). However, excessive and persistent inflammation is a driving force for many chronic diseases. In addition to obvious inflammatory diseases such as rheumatoid arthritis, it is well accepted that cancer, Alzheimer’s disease, and metabolic syndrome-related diseases like atherosclerosis, non-alcoholic fat liver disease, and diabetes mellitus type 2 are triggered by chronic low-grade inflammation (Kunnumakkara et al., 2018). As systemic inflammation is a complex process, this review refers only to inflammatory pathways that have been studied for chromanol and/or chromenol structures. Key regulatory factors and mediators of inflammatory processes in this context are receptors that sense proinflammatory stimuli, e.g. the toll-like receptors (TLRs), intracellular signaling molecules, like mitogen-activated protein kinases (MAPKs), and transcription factors, such as NF-κB or nuclear factor erythroid 2-related factor 2 (Nrf2). Further, enzymes that produce pro-inflammatory mediators such as prostaglandins (PGs) and leukotrienes (LTs) play a central role during the coordinated orchestra of the inflammatory process. This includes cyclooxygenases (COX) and lipoxygenases (LO). Other key players of inflammation are cytokines which are secreted by various cells and affect the interaction and communication between the different types of cells involved in inflammation (Aggarwal, 2009; Kunnumakkara et al., 2018). Important pro-inflammatory cytokines are interleukin (IL)-1β, IL-6, and IL-8 as well as tumor necrosis factor-α (TNF-α). Another important signaling molecule in inflammatory processes is nitric oxide (Aggarwal, 2009). In the following, chromanol and chromenol structures regulating the expression of key pro-inflammatory enzymes and the respective formation of signaling molecules are outlined.

#### Chromanols

A detailed overview on the biological activities of chromanols linked to inflammation is provided in **Table 1**.

**Table 1:** Overview on the biological activities of chromanols linked to inflammation.

Nitric oxide		Eicosanoid-mediated				Cytokine-mediated		
iNOS	NO	COX-2		PGE_2_		IL-1β	IL-6	TNF-α
**α-TOH**								
LPS iNOS PE RAW264.7 5 µM no inhibition (Wallert et al., 2015)	LPS NO PrD RAW264.7 20 µM no inhibition (Wallert et al., 2015)	LPS COX-2 PE, A RAW264.7 5 µM no inhibition (Wallert et al., 2015)	LPS COX-2 GE RAW264.7 100 µM no inhibition (Ciffolilli et al., 2015)	LPS PGE_2_ PrD RAW264.7 100 µM inhibition (Wallert et al., 2015)	IL-1β PGE_2_ PrD A549 cells 50 µM no inhibition (Jiang et al., 2008)	LPS pro IL-1β GE RAW264.7 100 µM inhibition (Wallert et al., 2015)	LPS IL-6 GE RAW264.7 100 µM no inhibition (Wallert et al., 2015)	LPS TNF-α GE RAW264.7 100 µM no inhibition (Wallert et al., 2015)
LPS iNOS PE RAW264.7 10 µM inhibition (Jiang et al., 2000)	LPS NO PrD RAW264.7 10 µM no inhibition (Jiang et al., 2000)	LPS COX E, A RAW264.7 50 µM no inhibition (Jiang et al., 2000)	LPS COX-2 A m_PM 500 ppm inhibition (Beharka et al., 2002)	LPS PGE_2_ PrD RAW264.7 50 µM inhibition (Jiang et al., 2000)	Age PGE_2_ PrD human 800 mg/d inhibition (Meydani et al., 1990)	LPS pro IL-1β GE RAW264.7 100 µM inhibition (Ciffolilli et al., 2015)	LPS IL-6 PrD RAW264.7 23 µM no inhibition (Yam et al., 2009)	LPS TNF-α PrD RAW264.7 23 µM induction (Yam et al., 2009)
LPS iNOS E RAW264.7 20 µM no inhibition (Schmölz et al., 2017)	LPS NO PrD RAW264.7 20 µM induction (Schmölz et al., 2017)	LPS COX-2 E RAW264.7 23 µM no inhibition (Yam et al., 2009)		LPS PGE_2_ PrD RAW264.7 23 µM induction (Yam et al., 2009)	LPS PGE_2_ PrD RAW264.7 100 µM inhibition (Ciffolilli et al., 2015)		LPS IL-6 GE RAW264.7 100 µM no inhibition (Ciffolilli et al., 2015)	LPS TNF-α GE RAW264.7 100 µM no inhibition (Ciffolilli et al., 2015)
LPS iNOS GE RAW264.7 100 µM no inhibition (Ciffolilli et al., 2015)	LPS NO PrD RAW264.7 100 µM no inhibition (Ciffolilli et al., 2015)			LPS PGE_2_ PrD m_PM 500 ppm inhibition (Beharka et al., 2002)				
	LPS NO PrD m_PM 500 ppm inhibition (Beharka et al., 2002)							
**β-TOH**								
				IL-1β PGE_2_ PrD A549 cells 50 µM no inhibition (Jiang et al., 2008)				
**γ-TOH**								
LPS iNOS PE RAW264.7 10 µM inhibition (Jiang et al., 2000)	LPS NO PrD RAW264.7 10 µM inhibition (Jiang et al., 2000)	LPS COX E + A RAW.264.7 50 µM no inhibition (Jiang et al., 2000)	IL-1β COX-2 A A549 cells 50 µM inhibition (Jiang et al., 2008)	LPS PGE_2_ PrD RAW264.7 50 µM inhibition (Jiang et al., 2000)	IL-1β PGE_2_ PrD A549 cells 25 µM inhibition (Jiang et al., 2008)		AOM IL-6 PrD BALB/c mice 0.1% of diet inhibition (Jiang et al., 2013)	
		IL-1β COX-2 PE A549 cells 40 µM no inhibition (Jiang et al., 2008)	LPS COX A A549 10 µM inhibition (Jiang et al., 2000)	IL-1β PGE_2_ PrD A549 cells 40 µM inhibition (Jiang et al., 2000)				
**δ-TOH**								
LPS iNOS E RAW264.7 20 µM inhibition (Schmölz et al., 2017)	LPS NO PrD RAW264.7 20 µM inhibition (Schmölz et al., 2017)	IL-1β COX-2 PE A549 cells 40 µM no inhibition (Jiang et al., 2008)	IL-1β COX-2 A A549 cells 50 µM inhibition (Jiang et al., 2008)	IL-1β PGE_2_ PrD A549 cells 25 µM inhibition (Jiang et al., 2008)				
**α-T3**								
	LPS NO PrD RAW264.7 23.5 µM inhibition (Yam et al., 2009)	IL-1β COX-2 PE A549 cells 10 µM no inhibition (Jiang et al., 2008)	LPS COX-2 PE RAW264.7 23.5 µM no inhibition (Yam et al., 2009)				LPS IL-6 PrD RAW264.7 23.5 µM inhibition (Yam et al., 2009)	LPS TNF-α PrD RAW264.7 23.5 µM inhibition (Yam et al., 2009)
		LPS COX-2 GE RAW264.7 23.5 µM inhibition (Yam et al., 2009)						
**γ-T3**								
	LPS NO PrD RAW264.7 24 µM inhibition (Yam et al., 2009)	LPS COX-2 GE RAW264.7 24 µM inhibition (Yam et al., 2009)	LPS COX-2 GE m_BMDM 1 µM inhibition (Kim et al., 2018)	LPS PGE_2_ PrD RAW264.7 24 µM inhibition (Yam et al., 2009)	LPS PGE_2_ PrD m_BMDM 1 µM inhibition (Kim et al., 2018)	LPS pro IL-1β GE/PrD m_BMDM 1 µM inhibition (Kim et al., 2018)	LPS IL-6 PrD RAW264.7 24 µM inhibition (Yam et al., 2009)	LPS TNF-α PrD RAW264.7 24 µM no inhibition (Yam et al., 2009)
		LPS COX-2 PE RAW264.7 24 µM no inhibition (Yam et al., 2009)				LPS pro IL-1β PrD m_BMDM 1 µM inhibition (Kim et al., 2016)	diabetes IL-6 PrD *db/db* mice 0.1% of diet inhibition (Kim et al., 2016)	diabetes TNF-α PrD *db/db* mice 0.1% of diet inhibition (Kim et al., 2016)
						LPS IL-1β PrD m_BMDM 1 µM inhibition (Kim et al., 2016)		
**δ-T3**								
	LPS NO PrD RAW264.7 25 µM inhibition (Yam et al., 2009)	LPS COX-2 GE RAW264.7 25 µM inhibition (Yam et al., 2009)	LPS COX-2 PE RAW264.7 25 µM no inhibition (Yam et al., 2009)	LPS PGE_2_ PrD RAW264.7 25 µM inhibition (Yam et al., 2009)			LPS IL-6 PrD RAW264.7 25 µM inhibition (Yam et al., 2009)	LPS TNF-α PrD RAW264.7 25 µM induction (Yam et al., 2009)
**α-T-13′-OH**								
LPS iNOS E RAW264.7 10 µM inhibition (Ciffolilli et al., 2015)	LPS NO PrD RAW264.7 10 µM inhibition (Ciffolilli et al., 2015)	LPS COX-2 E RAW264.7 10 µM inhibition (Ciffolilli et al., 2015)		LPS PGE_2_ PrD RAW264.7 10 µM inhibition (Ciffolilli et al., 2015)		LPS IL-1β PrD RAW264.7 10 µM inhibition (Ciffolilli et al., 2015)	LPS IL-6 PrD RAW264.7 10 µM inhibition (Ciffolilli et al., 2015)	LPS TNF-α PrD RAW264.7 10 µM no inhibition (Ciffolilli et al., 2015)
LPS iNOS E RAW264.7 10 µM inhibition (Schmölz et al., 2017)	LPS NO PrD RAW264.7 10 µM inhibition (Schmölz et al., 2017)							
**α-T-13′-COOH**								
LPS iNOS PE RAW264.7 5 µM inhibition (Wallert et al., 2015)	LPS NO PrD RAW264.7 5 µM inhibition (Wallert et al., 2015)	LPS COX-2 PE RAW264.7 5 µM inhibition (Wallert et al., 2015)	IL-1β COX-2 A platelet 10 µM inhibition (Pein et al., 2018)	LPS PGE_2_ PrD RAW264.7 5 µM inhibition (Wallert et al., 2015)	LPS PGE_2_ PrD h_monocytes 10 µM no inhibition (Pein et al., 2018)	LPS pro IL-1β GE RAW264.7 5 µM inhibition (Wallert et al., 2015)	LPS IL-6 GE RAW264.7 5 µM no inhibition (Wallert et al., 2015)	LPS TNF-α GE RAW264.7 5 µM no inhibition (Wallert et al., 2015)
LPS iNOS E RAW264.7 5 µM inhibition (Schmölz et al., 2017)	LPS NO PrD RAW264.7 5 µM inhibition (Schmölz et al., 2017)	LPS COX-2 A RAW264.7 5 µM no inhibition (Wallert et al., 2015)	– COX-2 A enzyme 10 µM no inhibition (Pein et al., 2018)					
**δ-T-13′-OH**								
LPS iNOS E RAW264.7 10 µM inhibition (Schmölz et al., 2017)	LPS NO PrD RAW264.7 10 µM inhibition (Schmölz et al., 2017)							
**δ-T-13′-COOH**								
LPS iNOS E RAW264.7 5 µM inhibition (Schmölz et al., 2017)	LPS NO PrD RAW264.7 5 µM inhibition (Schmölz et al., 2017)	IL-1β COX-2 A A549 4 µM inhibition (Jiang et al., 2008)	– COX-2 A enzyme 5 µM inhibition (Jang et al., 2016)					
		– COX-2 A enzyme 4 µM inhibition (Jiang et al., 2008)						
**δ-T-9′-COOH**								
		– COX-2 A enzyme 20 µM no inhibition (Jiang et al., 2008)	IL-1β COX-2 A A549 6 µM inhibition (Jiang et al., 2008)					
**α-T-5′-COOH**								
		– COX-2 A enzyme 140 µM inhibition (Jiang et al., 2008)						
**α-T-3′-COOH**								
TNF-α iNOS PE EOC-20 cells 100 µM inhibition (Grammas et al., 2004)	TNF-α NO PrD RAEC cells 100 µM inhibition (Grammas et al., 2004)			LPS PGE_2_ PrD RAEC cells 100 µM inhibition (Grammas et al., 2004)	LPS PGE_2_ PrD EOC-20 cells 100 µM inhibition (Grammas et al., 2004)			
	TNF-α/LPS NO PrD EOC-20 cells 100 µM inhibition (Grammas et al., 2004)							
**γ-T-3′-COOH**								
TNF-α iNOS E EOC-20 cells 100 µM inhibition (Grammas et al., 2004)	LPS NO PrD RAW264.7 10 µM no inhibition (Jiang et al., 2000)	IL-1β COX-2 A A549 50 µM inhibition (Jiang et al., 2000)	– COX-2 A enzyme 450 µM inhibition (Jiang et al., 2008)	LPS PGE_2_ PrD RAW264.7 10 µM inhibition (Jiang et al., 2000)	IL-1β PGE_2_ PrD A549 40 µM inhibition (Jiang et al., 2000)			
	LPS NO PrD EOC-20 cells 100 µM inhibition (Grammas et al., 2004)	IL-1β COX-2 PE A549 50 µM no inhibition (Jiang et al., 2000)						
**δ-T3-13′-COOH**								
LPS iNOS E RAW264.7 5 µM inhibition (Wallert et al., 2019)	LPS NO PrD RAW264.7 5 µM inhibition (Wallert et al., 2019)	– COX-2 A enzyme 9.8 µM inhibition (Jang et al., 2016)	LPS COX-2 A h_monocytes 10 µM no inhibition (Pein et al., 2018)	LPS PGE_2_ PrD RAW264.7 5 µM inhibition (Wallert et al., 2019)	LPS PGE_2_ PrD h_monocytes 300 nM inhibition (Pein et al., 2018)	LPS pro IL-1β GE RAW264.7 5 µM inhibition (Wallert et al., 2019)	LPS IL-6 GE RAW264.7 5 µM inhibition (Wallert et al., 2019)	LPS TNF-α GE RAW264.7 5 µM inhibition (Wallert et al., 2019)
		LPS COX-2 E RAW264.7 5 µM inhibition (Wallert et al., 2019)		HFD PGE_2_ PrD m_APOE^-/-^ 1 mg/kg no inhibition (Wallert et al., 2019)		HFD IL-1β PrD m_APOE^-/-^ 1 mg/kg no inhibition (Wallert et al., 2019)		
**SCA D**								
LPS iNOS PE RAW264.7 15 µM inhibition (Heo et al., 2014)	LPS NO PrD RAW264.7 15 µM inhibition (Heo et al., 2014)	LPS COX-2 PE RAW264.7 15 µM inhibition (Heo et al., 2014)		LPS PGE_2_ PrD RAW264.7 15 µM inhibition (Heo et al., 2014)		LPS IL-1β PrD RAW264.7 60 µM inhibition (Heo et al., 2014)	LPS IL-6 PrD RAW264.7 30 µM inhibition (Heo et al., 2014)	LPS TNF-α PrD RAW264.7 60 µM inhibition (Heo et al., 2014)
**SCA E**								
LPS iNOS PE RAW264.7 29 µM inhibition (Lee et al., 2013)	LPS NO PrD RAW264.7 29 µM inhibition (Lee et al., 2013)	LPS COX-2 PE RAW264.7 29 µM inhibition (Lee et al., 2013)		LPS PGE_2_ PrD RAW264.7 29 µM inhibition (Lee et al., 2013)		LPS IL-1β PrD RAW264.7 12 µM inhibition (Lee et al., 2013)		LPS TNF-α PrD RAW264.7 29 µM inhibition (Lee et al., 2013)
**SCA G**								
LPS iNOS PE RAW264.7 10 µM inhibition (Yoon et al., 2012a)	LPS NO PrD RAW264.7 10 µM inhibition (Yoon et al., 2012a)	LPS COX-2 PE RAW264.7 10 µM inhibition (Yoon et al., 2012a)		LPS PGE_2_ PrD RAW264.7 10 µM inhibition (Yoon et al., 2012a)		LPS IL-1β PrD RAW264.7 10 µM inhibition (Yoon et al., 2012a)	LPS IL-6 PrD RAW264.7 10 µM inhibition (Yoon et al., 2012a)	LPS TNF-α PrD RAW264.7 10 µM inhibition (Yoon et al., 2012a)
**δ-AC**								
						LPS IL-1β PrD monocytes 1 µM inhibition (Richomme et al., 2017)		LPS TNF-α PrD monocytes 10 µM inhibition (Richomme et al., 2017)

The effects of the respective compounds on inflammation have been divided into activities mediated by nitric oxide (iNOS, NO), eicosanoids (COX-2, PGE_2_), and cytokines (IL-1β, IL-6, TNF-α). The content of each cell of the table is constructed as follows (read from top to bottom): (i) used stimulus; (ii) investigated parameter; (iii) cell type, tissue, mouse, or other models used for investigation; (iv) used concentration of the respective compound; (v) observed effect on the studied parameter; (vi) reference. When no stimulus was used or was required for the studies, the respective row is marked with a dash. The following abbreviations are used: A, activity; A549, human adenocarcinoma alveolar basal epithelial cells; BALB/c mice, albino laboratory-bred strain of the house mouse; Apoe^-/-^ mice, apolipoprotein E deficient mice; BMDM, bone marrow derived macrophages; COX-2, cyclooxygenase 2; EOC-20, epithelial ovarian cancer cells; E, expression; GE, gene expression; HFD, high-fat diet; h, human; iNOS, inducible nitric oxide synthase; IL, interleukin; db/db mice, leptin receptor activity deficient mice; LPS, lipopolysaccharides; m, murine; RAW264.7, macrophages derived from abelson murine leukemia virus-induced tumor; NO, nitric oxide; ppm, parts per million; PM, peritoneal macrophages; PrD, production; PGE_2_, prostaglandin E_2_; PE, protein expression; RAEC, rat aortic endothelial cells; TNF-α, tumor necrosis factor α.

All results obtained from in vivo studies are marked in gray.

##### Tocopherols and Tocotrienols

Data available for TOHs and T3s correlate with their abundance in humans. Therefore, α- and γ-TOH as well as their respective T3 forms were mostly investigated so far. α-Tocopherol is regarded as the only form within the group of vitamin E that has been shown to mediate actual vitamin E function (Azzi, 2019). Further, α-TOH is considered as the most abundant vitamin E form in human nutrition, followed by γ-TOH. Relevance of T3s as anti-inflammatory compounds has just recently come to fore of research and will be presented in the following sections.

###### Tocopherols

For many years, TOHs were solely known for their anti-oxidative capacity. However, Azzi and colleagues discovered additional gene regulatory effects of α-TOH that are independent from its capacity as an antioxidant. α-TOH revealed distinct effects on nitric oxide- and eicosanoid-mediated inflammation. For instance, α-TOH (10 µM) decreased the expression level of inducible nitric oxide synthase (iNOS) in LPS-stimulated murine RAW264.7 macrophages (Jiang et al., 2000). However, others could not confirm the observed alteration of iNOS expression using 5 µM (Wallert et al., 2015), 20 µM (Schmölz et al., 2017), or even 100 µM (Ciffolilli et al., 2015) α-TOH. In line with this, iNOS-mediated formation of nitric oxide remained unchanged in RAW264.7 macrophages by coincubation with α-TOH (Jiang et al., 2000; Ciffolilli et al., 2015; Wallert et al., 2015). In contrast, the formation of PGE_2_ was blocked by 23 to 100 µM α-TOH in LPS-stimulated RAW264.7 macrophages (Jiang et al., 2000; Yam et al., 2009; Ciffolilli et al., 2015; Wallert et al., 2015), but not in IL-1β-stimulated A549 epithelial cells (Jiang et al., 2008). Unexpectedly, upstream-regulated COX-2 expression and activity remained unchanged in RAW264.7 macrophages at concentrations of 23 to 100 µM α-TOH. Furthermore, cytokine-mediated inflammation was not regulated by α-TOH (Yam et al., 2009), except for an inhibition of IL-1β gene expression in RAW264.7 macrophages using 100 µM (Ciffolilli et al., 2015; Wallert et al., 2015). Beside external stimuli, induction of inflammation, mainly *via* the TLR4-NF-κB signaling pathway, senescence of cells, and aging are also known triggers of inflammation (Lasry and Ben-Neriah, 2015). Indeed, 24-months-old mice are characterized by an increased inflammatory state compared to younger mice (six months). Application of 500 ppm α-TOH acetate lowered aging-induced increases of nitric oxide and PGE_2_ plasma levels as well as COX-2 activity compared to 24-months-old mice fed 30 ppm (Beharka et al., 2002). In line with this, supplementation with 800 mg α-TOH/kg/d in elder humans for 30 days lead to significantly lower levels of PGE_2_ in plasma and peripheral blood mononuclear cells compared to vehicle-treated controls (Meydani et al., 1990).

The second most abundant form of vitamin E, γ-TOH, is more prominent for its anti-inflammatory capacity compared to α-TOH. Release of nitric oxide by LPS-stimulated RAW264.7 cells was significantly inhibited using 10 µM γ-TOH (Jiang et al., 2000). Release of eicosanoids inflammation, more precisely PGE_2_, in LPS-stimulated RAW264.7 cells and in IL-1β-stimulated A549 cells was inhibited by 10 µM (IC_50_ 7.5 µM) (Jiang et al., 2000) and 25–40 µM (IC_50_ 4–7 µM) (Jiang et al., 2000; Jiang et al., 2008), respectively. However, COX-2 expression (Jiang et al., 2000; Jiang et al., 2008) and activity (Jiang et al., 2000; Jang et al., 2016) remained unchanged in LPS-stimulated RAW264.7 macrophages, whereas COX-2 activity was inhibited by 50 µM γ-TOH in IL-1β-stimulated A549 epithelial cells (Jiang et al., 2008). Azoxymethane-induced IL-6 production was dampened in BALB/c mice by a γ-TOH-enriched diet (Jiang et al., 2013).

δ-tocopherol (20 µM) significantly decreased LPS-induced expression of iNOS (by 60% at mRNA and by 48% at protein level) and formation of nitric oxide (by 36%) in RAW264.7 macrophages (Schmölz et al., 2017). Jiang et al. reported an inhibition of COX-2 activity, but not COX-2 expression in IL-1β-stimulated A549 cells (Jiang et al., 2008), whereas Jang et al. did not observe altered COX-2 activity after δ-TOH treatment using a human recombinant enzyme-based assay (Jang et al., 2016). However, formation of PGE_2_ was significantly blocked (IC_50_ 1–3 µM) (Jiang et al., 2008). The least abundant form of tocopherols, β-TOH has been rarely studied regarding its anti-inflammatory capacity. Studies available so far did not reveal any anti-inflammatory effects of β-TOH (Jiang et al., 2008).

###### Tocotrienols

Recent publications reported a more pronounced anti-inflammatory capacity of T3s compared to TOHs, with γ-T3 and α-T3 showing the strongest effects. α-, δ-, and γ-T3 significantly decreased LPS-mediated formation of nitric oxide (by 11%, 31%, 19%, respectively) and PGE_2_ (by 30%, 55%, 20%, respectively) in RAW264.7 macrophages treated with 23.5 µM of the respective compound (Yam et al., 2009) as well as bone marrow-derived macrophages (BMDMs) using 1 µM of γ-T3 (Kim et al., 2018). Expression of COX-2 mRNA was inhibited by α-, δ-, and γ-T3, whereas protein expression remained unchanged (Jiang et al., 2008; Yam et al., 2009; Kim et al., 2018). In addition, cytokine-driven inflammation is also dampened by α-, δ-, and γ-T3, which reduced the release of IL-6 and TNF-α in LPS-stimulated RAW264.7 cells. However, γ-T3 reduced expression of IL-6 and TNF-α mRNA as well as the secretion of IL-6, but not of TNF-α in this cell model (Yam et al., 2009). Furthermore, first reports suggest inhibitory effects of γ-T3 on the NLR family pyrin domain containing 3 (NLRP3) inflammasome. In brief, 1 µM γ-T3 suppressed mRNA expression of pro-IL-1β and -18 as well as respective formation of active IL-1β and -18. This has been observed in LPS/nigericin- as well as LPS/palmitate-stimulated BMDMs and *db/db* mice fed with a diet containing 0.1% γ-T3 for eight weeks (Kim et al., 2016; Kim et al., 2018).

##### Metabolites of Tocopherols and Tocotrienols

We here present a report on selected structures formed during hepatic catabolism of vitamin E, for which data on the biological activity was available. Metabolites formed during physiological hepatic metabolism of vitamin E are highly potent anti-inflammatory compounds with different efficiencies, depending on their methylation pattern (Azzi, 2019) and the number of isoprene units forming the side-chain (Schmölz et al., 2017). Metabolism of non-α-TOH forms of vitamin E is more pronounced, resulting from the lower affinities of these molecules to the α-tocopherol transfer protein. However, α-metabolites revealed significant anti-inflammatory properties. The most widely studied metabolites are the LCMs α-T-13′-OH and -COOH and the short-chain metabolites α- and γ-3′-T-COOH, likely due to their presence in plasma, feces, and urine, respectively, which may account for their physiological relevance (Jiang et al., 2007).

###### Long- and Intermediate-Chain Tocopherol-Derived Metabolites

Birringer and coworkers showed the relevance of the terminal oxidative modification of the side-chain for biological activity (Birringer et al., 2018). During the hepatic metabolism of TOHs, T-13′-OH are the first metabolites that are formed; these LCMs show distinct effects that are different from those of their respective metabolic precursor (for details, see **Chapter 2.1.1. *Tocopherols and Tocotrienols***). Both, α- and δ-T-13′-OH significantly decreased mRNA (29–72% and 87%, respectively) and protein (40–53% and 53%, respectively) expression of iNOS and the production of nitric oxide (56–69% and 49%, respectively) in LPS-stimulated murine RAW264.7 macrophages at a concentration of 10 µM, thus showing comparable effect sizes independent from the methylation pattern of the chromanol ring system (Ciffolilli et al., 2015; Schmölz et al., 2017). Furthermore, α-T-13′-OH significantly decreased expression of COX-2 mRNA and protein (64% and 49%, respectively), IL-1β (64%) and IL-6 (68%) mRNA, and the production of PGE_2_ (55%) (Ciffolilli et al., 2015).

Notably, the length of the side-chain is important for the mediation of anti-inflammatory effects. Accordingly, both α-T-13′-COOH (5 µM) and δ-T-13′-COOH (5 µM) significantly decreased expressions of iNOS and COX-2 mRNAs as well as proteins in murine LPS-stimulated RAW264.7 macrophages (Wallert et al., 2015; Schmölz et al., 2017). Further, δ-T-13′-COOH inhibited the activity of purified recombinant COX-2 enzyme (5 µM [Jiang et al., 2008; Jang et al., 2016]) as well as in human lung adenocarcinoma A549 cells (4 µM, [Jiang et al., 2008)). Interestingly, the activity of recombinant COX-2 enzymes remained unchanged by α-T-13′-COOH (5–10 µM) (Wallert et al., 2015; Pein et al., 2018). LPS-induced production of the respective signaling molecules, nitric oxide and PGE_2_, was completely blocked in murine macrophages (5 µM), but not in LPS-activated human primary monocytes (10 µM) (Pein et al., 2018). In addition, 5-LO-induced formation of pro-inflammatory leukotrienes was dampened by α-T-13′-COOH in LPS-stimulated monocytes (LTB_4_), activated human neutrophils, activated human blood, zymosan-induced mouse peritonitis (LTC4), as measured in plasma and exudate, and ovalbumin-induced bronchial hyperreactivity in mice (Pein et al., 2018). Effective concentrations of α-T-13′-COOH, that inhibit 5-LO product formation *in vitro*, were in a range that was detected for the metabolite in human and mice serum without supplementation (<0.3 µM). Furthermore, expression of pro-IL-1β was down-regulated by 5 µM α-T-13′-COOH, whereas IL-6 and TNF-α remained unchanged (Wallert et al., 2015).

Degradation of the LCMs of different vitamin E forms results in formation of respective ICMs that are further processed to SCMs. These metabolic end-products do not accumulate in plasma or tissues and their physiological relevance is therefore considered as less important. Hence, data on these metabolites are scarce. To date, anti-inflammatory effects, *i.e.* the inhibition of COX-2 activity (IC_50_ 6 µM), by δ-9′-T-COOH have been reported in human lung adenocarcinoma A549 cells (Jiang et al., 2008).

###### Long- and Intermediate-Chain Tocotrienol-Derived Metabolites

Within the group of T3-derived metabolites, the LCM δ-T3-13′-COOH (*i.e*. garcinoic acid) is the most potent anti-inflammatory compound of the ones studied so far. Expression of iNOS (by 97%), COX-2 (by 70%), pro-IL-1β (by 61%), IL-6 (by 70%), and TNF-α (by 25%) mRNA was decreased by 5 µM δ-T3-13′-COOH in LPS-stimulated murine RAW264.7 macrophages. Consequently, protein expression of iNOS (by 83%), COX-2 (by 33%), and the respective formation of NO (by 81%), PGE_2_ (by 90%) and thromboxane (TX)B_2_ (by 91%) were dampened in LPS-stimulated murine RAW264.7 macrophages (Wallert et al., 2019). Formation of PGE_2_ in LPS-stimulated monocytes was inhibited already by 300 nM δ-T3-13′-COOH (Pein et al., 2018). In line with this, δ-T3-13′-COOH also inhibited activity of microsomal PGE_2_ synthase (by nearly 70%) at a concentration of 10 μM in a cell-free assay using microsomes of IL-1β-stimulated human lung adenocarcinoma A549 cells as an enzyme source (Alsabil et al., 2016; Pein et al., 2018). However, in *Apoe* ^-/-^ mice fed a high fat diet with 1 mg/kg δ-T3-13′-COOH for 8 weeks neither nitric oxide, PGE_2_, TXB_2_ nor IL-1β concentrations in plasma were altered compared to the control group (Wallert et al., 2019). However, contrary data exist also for the alteration of prostaglandins following inhibition of COX-2 activity: IC_50_ 9.8 µM (Jang et al., 2016) and IC_50_ >10 µM (Pein et al., 2018).

###### Short-Chain Tocopherol-Derived Metabolites

5′-T-COOH (CMBHC) and 3′-T-COOH (CEHC) are the SCMs. Physiologically formed γ-3′-T-COOH was mainly detected in urine. Supplementation of α-TOH enhances the hepatic metabolism of α-TOH, which in turn increases degradation of α-TOH and excretion of α-5′-T-COOH and α-3′-T-COOH *via* urine. Both, α-5′-T-COOH (IC_50_ 140 µM) and γ-3′-T-COOH (IC_50_ 450 µM) showed marginal inhibitory effects on human recombinant COX-2 activity (Jiang et al., 2008). However, in IL-1β-stimulated A549 cells, γ-3′-T-COOH (50 µM) exhibited stronger inhibition of COX-2 activity. Formation of PGE_2_ was also inhibited in IL-1β-stimulated A549 (50 µM), LPS-stimulated RAW264.7 (10 µM), as well as TNF-α-stimulated RAEC (IC_50_ 59 μM) and EOC-20 cells (IC_50_ 66 µM) (Jiang et al., 2000; Grammas et al., 2004). The TNF-α-induced release of nitric oxide was blocked in EOC-20 (IC_50_ 58 µM) and RAEC cells (IC_50_ 56 µM) by α-3′-T-COOH, whereas 100 µM γ-3′-T-COOH inhibited production of nitric oxide in EOC-20 cells by 10% only (Grammas et al., 2004). In contrast, both α-3′-T-COOH and γ-3′-T-COOH decreased production of nitric oxide in LPS-stimulated EOC-20 cells (Grammas et al., 2004). Notably, lower concentrations did not alter production of nitric oxide (Jiang et al., 2000; Grammas et al., 2004).

##### Sargachromanols

The sargachromanol forms D, E, and G isolated from *Sargassum siliquastrum* also exert anti-inflammatory effects in LPS-stimulated RAW264.7 macrophages in a concentration-dependent manner. Sargachromanol forms D, E, and G inhibited expression of iNOS protein to 30–50% with concentrations of 15, 12.5, and 20 µM, respectively. In contrast, inhibitory effects on the formation of the respective signaling molecule nitric varies compound-dependent between 10 and 90% (Lee et al., 2013), with SCA E being the most effective (Yoon et al., 2012a; Lee et al., 2013; Heo et al., 2014). Within the inflammatory eicosanoid pathway, expression of COX-2 was inhibited by 15% by SCA D and G and up to 90% by SCA E. The IC_50_ for the formation of COX-2-derived PGE_2_ was 15 µM (SCA D [Heo et al., 2014]), 12.5 µM (SCA E [Lee et al., 2013]), and 20 µM (SCA G [Yoon et al., 2012a]), respectively. The LPS-induced production of TNF-α, IL-6 and IL-1β was effectively blocked by SCA D (IC_50_ >60, >20–25, and 40 µM, respectively [Heo et al., 2014]), E (IC_50_ >25 µM, not investigated and >15 µM, respectively [Lee et al., 2013]), and G (IC_50_ 40, 20, and 20 µM, respectively [Yoon et al., 2012a]). The total inflammatory capacity, as determined by the expression of iNOS and COX-2, the production of their respective signaling molecules, nitric oxide and PGE_2_, as well as the production of cytokines leads to the following estimation of compound effectiveness: SCA E > D > G.

##### Amplexichromanols

Amplexichromanols can be distinguished as α-, β-, γ-, δ-forms. δ-Amplexichromanols have been shown to inhibit the secretion of TNF-α (IC_50_ <10 µM) and IL-1β (IC_50_ 10 µM) in LPS-stimulated monocytes (Richomme et al., 2017). To the best of our knowledge, there are no reports on anti-inflammatory effects of the other forms of AC.

#### Chromenols

Compared to the complex group of structures comprising the chromanol family, chromenol structures are less ubiquitous. Sargachromenol is described here as a representative of the chromenols with anti-inflammatory effects. An ethanolic extract of *Myagropsis myagroides* inhibited nitric oxide-, eicosanoid-, and cytokine-mediated pathways and the inflammatory response (**Table 2**), with sargachromenol being the lead compound in the extract (Kim et al., 2014). Further studies using isolated sargachromenol from different sources confirmed the results obtained by Kim et al. For instance, sargachromanol isolated from the marine brown alga *Sargassum serratifolium* inhibited peroxinitrite anion-mediated albumin nitration with an IC_50_ of 5 µM (Ali et al., 2017). Furthermore, the COX-2 pathway was inhibited using 50 µM and 100 ppm sargachromenol isolated from *Sargassum micracanthum* (Yang et al., 2013) and *Iryanthera juruensis* seeds (Silva et al., 2007), respectively. Here, the effect sizes of 70 and 84% found by Yang *et al*. and Silva *et al.*, respectively, are comparable with respect to the inhibition of the expression of COX-2 protein. For the respective signaling molecule PGE_2_ an IC_50_ value of 30 µM was defined (Yang et al., 2013). In addition, inhibitory effects were observed for the expression of iNOS protein (95%) and the formation of nitric oxide (IC_50_ 82 µM) (Yang et al., 2013).

**Table 2:** Overview on the biological activities of chromenols linked to inflammation.

Nitric oxide		Eicosanoid-mediated		Cytokine-mediated		
iNOS	NO	COX-2	PGE2	IL-1β	IL-6	TNF-α
**Sargachromenol**						
LPS iNOS PE BV-2 cells 2.7 µM inhibition (Kim et al., 2014)	LPS NO PrD BV-2 cells 2.7 µM inhibition (Kim et al., 2014)	LPS COX-2 E BV-2 cells 2.7 µM inhibition (Kim et al., 2014)	LPS PGE_2_ PrD BV-2 cells 2.7 µM inhibition (Kim et al., 2014)	LPS IL-1β PrD BV-2 cells 2.7 µM inhibition (Kim et al., 2014)	LPS IL-6 PrD BV-2 cells 2.7 µM inhibition (Kim et al., 2014)	LPS TNF-α PrD BV-2 cells 2.7 µM inhibition (Kim et al., 2014)
LPS iNOS PE RAW264.7 50 µM inhibition (Yang et al., 2013)	LPS NO PrD RAW264.7 50 µM inhibition (Yang et al., 2013)	LPS COX-2 PE RAW264.7 50 µM inhibition (Yang et al., 2013)	LPS PGE_2_ PrD RAW264.7 50 µM inhibition (Yang et al., 2013)			
	peroxynitrite NO PrD BSA nitrition 2.5 µM inhibition (Ali et al., 2017)	– COX-2 A enzyme 100 ppm inhibition (Silva et al., 2007)				

The effects of the respective compounds on inflammation have been divided into activities mediated by nitric oxide (iNOs, NO), eicosanoids (COX-2, PGE_2_), and cytokines (IL-1β, IL-6, TNF-α). The content of each cell of the table is constructed as follows (read from top to bottom): (i) used stimulus; (ii) investigated parameter; (iii) cell type or other models used for investigation; (iv) used concentration of the respective compound; (v) observed effect on the studied parameter; (vi) reference. In the publications where no stimulus was used or was required for the studies, the respective row is marked with a dash. The following abbreviations are used: A, activity; BSA, bovine serum albumin; BV-2, brain microglial cells transformed by recombinant retrovirus (v-raf/v-mic); COX-2, Cyclooxygenase 2; E, expression; iNOS, induce able nitric oxide synthase; IL-1β, interleukin 1β; IL-6, interleukin 6; LPS, lipopolysaccharides; m, murine; RAW264.7, macrophages derived from abelson murine leukemia virus-induced tumor; NO, nitric oxide; PrD, production; PGE_2_, prostaglandin E_2_; PE, protein expression; TNF-α, tumor necrosis factor α.

### Carcinogenesis

For the evaluation of anti-carcinogenic effects of chromanol and chromenol structures, key apoptotic pathways, such as cleavage of poly-[ADP-ribose]-polymerase 1 (PARP-1), caspases 3, 7, 8, and 9 as well as anti-proliferative and cytotoxic properties on cancer cell lines and further markers of carcinogenesis marker in mice were evaluated (**Figure 7**). In addition, large-scaled human trials investigating preventive and therapeutic effects of some tested compounds will be discussed in the following chapter.

#### Chromanols

A detailed overview on the biological activities of chromanols linked to carcinogenesis is provided in **Table 3**.

**Table 3:** Overview on the biological activities of chromanols linked to carcinogenesis.

Apoptosis/Necrosis mediated					Proliferation		Viability	
PARP-1	Casp8	Casp9	Casp3	Casp7				
**α-TOH**								
PARP-1 CL MDA‐MB‐231 MCF-7 cells 23 µM no induction (Loganathan et al., 2013)	Casp8 A MiaPaCa-2 50 µM no induction (Husain et al., 2011)		Casp3 A MiaPaCa-2 50 µM no induction (Husain et al., 2011)	Casp7 CL SW 480 cells HCT-116 100 µM no induction (Campbell et al., 2006)	MDA-MB-435 > 2000 µM MCF-7 cells 290 µM no inhibiton (Guthrie et al., 1997)	MDA-MB-435 230 µM no inhibition (Nesaretnam et al., 1995)		h_cc cells 200 µM no reduction (Campbell et al., 2006)
PARP-1 CL SW 480 cells HCT-116 100 µM no induction (Campbell et al., 2006)	Casp8 CL SW 480 cells HCT-116 100 µM no induction (Campbell et al., 2006)		Casp3 CL SW 480 cells HCT-116 100 µM no induction (Campbell et al., 2006)		m_NB2A cells inhibition h_ SaOs-2 cells no inhibition 50 µM (Azzi et al., 1993)	Du-145 cells LNCaP cells CaCo-2 cells 25 µM inhibition SaOs-2 cells no inhibition (Gysin et al., 2002)		MCF-7, MCF-7-C3 50 µM no reduction (Birringer et al., 2003)
					PC-3 HTB-82 50 µM inhibition (Galli et al., 2004)	MCF-7 cells 23 µM no inhibition (Nesaretnam et al., 1998)		
					MDA-MB-231 MCF-7 cells 46.5 µM no inhibition (Loganathan et al., 2013)	HT-29 100 µM inhibition (Campbell et al., 2006)		
**β-TOH**								
					Du-145 cells LNCaP cells SaOs-2 cells 25 µM inhibition (Gysin et al., 2002)	m_NB2A cells h_ SaOs-2 cells 50 µM no inhibition (Azzi et al., 1993)		MCF-7, MCF-7-C3 50 µM no reduction (Birringer et al., 2003)
**γ-TOH**								
PARP-1 CL SW 480 cells HCT-116 100 µM induction (Campbell et al., 2006)	Casp8 CL SW 480 cells HCT-116 100 µM induction (Campbell et al., 2006)		Casp3 CL SW 480 cells HCT-116 100 µM induction (Campbell et al., 2006)	Casp7 CL SW 480 cells HCT-116 100 µM induction (Campbell et al., 2006)	PC-3 cells HTB-82 cells 1 µM inhibition (Galli et al., 2004)	h_cc cells 100 µM inhibition (Campbell et al., 2006)	HCT-116, HT-29 50 µM no inhibition (Jang et al., 2016)	h_cc cells 100 µM reduction (Campbell et al., 2006)
					Du-145 cells LNCaP cells CaCo-2 cells SaOs-2 cells 25 µM inhibition (Gysin et al., 2002)	tumor count m_BALB/c 0.1% diet reduction (Jiang et al., 2013)	PC-3, LNCaP 50 µM inhibition (Jiang et al., 2012)	MCF-7, MCF-7-C3 50 µM no reduction (Birringer et al., 2003)
**δ-TOH**								
							MCF-7, MCF-7-C3 50 µM no reduction (Birringer et al., 2003)	HCT-116 inhibition HT-29 no reduction 50 µM (Jang et al., 2016)
**α-T3**								
PARP-1 CL, MDA‐MB‐231, MCF-7 cells 23.5 µM induction (Loganathan et al., 2013)	Casp8 A MiaPaCa-2 50 µM induction (Husain et al., 2011)		Casp3 A MiaPaCa-2 50 µM induction (Husain et al., 2011)		MDA-MB-435 211.9 µM MCF-7 cells 14.1 µM inhibition (Guthrie et al., 1997)	m_B16(F10) 110 µM inhibition (He et al., 1997)	SCID mice 200 mg/kg no reduction (Husain et al., 2011)	MiaPaCa-2, 50 µM no reduction (Husain et al., 2011)
PARP-1 CL MiaPaCa-2 50 µM no induction (Husain et al., 2011)					MDA-MB-231 22.5 µM MCF-7 cells 26.1 µM inhibition (Loganathan et al., 2013)	MCF-7 23.5 µM no inhibition (Nesaretnam et al., 1998)		MCF-7, MCF-7-C3 50 µM no reduction (Birringer et al., 2003)
**β-T3**								
								MiaPaCa-2, 50 µM reduction (Husain et al., 2011)
**γ-T3**								
PARP-1 CL, MDA‐MB‐231, MCF-7 cells 24.2 µM induction (Loganathan et al., 2013)	Casp8 A MiaPaCa-2 50 µM induction (Husain et al., 2011)	Casp9 CL PC-3, LNCaP 20 µM induction (Jiang et al., 2012)	Casp3 A MCF-7, MCF-7-C3 50 µM induction (Birringer et al., 2003)	Casp7 CL PC-3, LNCap 30/90 µM induction (Yap et al., 2008)	SKBR3, BT474 5 µM inhibition (Alawin et al., 2016)	rh_RLh-84 50 µM inhibition (Sakai et al., 2004)	MiaPaCa-2, 50 µM reduction (Husain et al., 2011)	PC-3, LNCaP 20 µM reduction (Jiang et al., 2012)
PARP-1 CL MiaPaCa-2 50 µM induction (Husain et al., 2011)	Casp8 CL PC-3, LNCap 30/90 µM induction (Yap et al., 2008)	Casp9 CL PC-3, LNCap 30/90 µM induction (Yap et al., 2008)	Casp3 A MiaPaCa-2 50 µM induction (Husain et al., 2011)		m_B16(F10) 20 µM inhibition (He et al., 1997)	PC-3 32 µM inhibition (Yap et al., 2008)		
PARP-1 CL PC-3, LNCaP 20 µM induction (Jiang et al., 2012)	Casp8 CL rh_RLh-84 25 µM induction (Sakai et al., 2004)		Casp3 CL PC-3, LNCap 30/90 µM induction (Yap et al., 2008)		MDA-MB-231 11.4 µM MCF-7 cells 15.4 µM inhibition (Loganathan et al., 2013)	MCF-7 14.6 µM inhibition (Nesaretnam et al., 1998)		
PARP-1 CL PC-3, LNCap 30/90 µM induction (Yap et al., 2008)			Casp3 CL rh_RLh-84 25 µM induction (Sakai et al., 2004)		MDA-MB-435 73.2 µM MCF-7 cells 4.9 µM inhibition (Guthrie et al., 1997)			
**δ-T3**								
PARP-1 CL, MDA‐MB‐231, MCF-7 cells 25.2 µM induction (Loganathan et al., 2013)	Casp8 A MiaPaCa-2 50 µM induction (Husain et al., 2011)		Casp3 A MiaPaCa-2 50 µM induction (Husain et al., 2011)		MDA-MB-435 226.8 µM MCF-7 cells 5 µM inhibition (Guthrie et al., 1997)	PC-3 41 µM LNCap 75 µM inhibition (Yap et al., 2008)		MiaPaCa-2, 50 µM reduction (Husain et al., 2011)
PARP-1 CL MiaPaCa-2 50 µM induction (Husain et al., 2011)					m_B16(F10) 10 µM inhibition (He et al., 1997)	MCF-7 25.2 µM inhibition (Nesaretnam et al., 1998)		
					MDA-MB-231 MCF-7 cells 17 µM inhibition (Loganathan et al., 2013)			
**α-T-13'-OH** (tocopherol derived)								
PARP-1 CL HepG2 cells 20 µM no induction (Birringer et al., 2010)		Casp9 CL HepG2 cells 20 µM no induction (Birringer et al., 2010)	Casp3 CL HepG2 cells 20 µM no induction (Birringer et al., 2010)	Casp7 CL HepG2 cells 20 µM no induction (Birringer et al., 2010)			m_C6 cells 10 µM reduction (Mazzini et al., 2009)	THP-1 ΜΦ 100 µM no reduction (Wallert et al., 2014a)
**α-T-13'-COOH** (tocopherol derived)								
PARP-1 CL HepG2 cells 20 µM induction (Birringer et al., 2010)		Casp9 CL HepG2 cells 20 µM induction (Birringer et al., 2010)	Casp3 CL HepG2 cells 20 µM induction (Birringer et al., 2010)	Casp7 CL HepG2 cells 20 µM induction (Birringer et al., 2010)			THP-1 ΜΦ 7.4 µM reduction (Wallert et al., 2014a)	HepG2 cells 13.5 µM reduction (Birringer et al., 2010)
**δ-T-13'-OH** (tocopherol derived)								
PARP-1 CL HepG2 cells 20 µM induction (Birringer et al., 2010)		Casp9 CL HepG2 cells 20 µM induction (Birringer et al., 2010)	Casp3 CL HepG2 cells 20 µM no induction (Birringer et al., 2010)	Casp7 CL HepG2 cells 20 µM induction (Birringer et al., 2010)			THP-1 100 µM no reduction (Schmölz et al., 2017)	HepG2 cells 50 µM no reduction (Birringer et al., 2010)
**δ-T-13'-COOH** (tocopherol derived)								
PARP-1 CL HepG2 cells 20 µM induction (Birringer et al., 2010)		Casp9 CL HepG2 cells 20 µM induction (Birringer et al., 2010)	Casp3 CL HepG2 cells 20 µM induction (Birringer et al., 2010)	Casp7 CL HepG2 cells 20 µM induction (Birringer et al., 2010)			HCT-116 HT-29 8.9/8.6 µM reduction (Jang et al., 2016)	HepG2 cells 6.5 µM reduction (Birringer et al., 2010)
PARP-1 CL HCT-116 20 µM induction (Jang et al., 2016)		Casp9 CL HCT-116 20 µM induction (Jang et al., 2016)					m_C6 cells 10 µM reduction (Mazzini et al., 2009)	HCT-116 HT-29 8.9/8.6 µM reduction (Jang et al., 2016)
							THP-1 11.1 µM reduction (Schmölz et al., 2017)	
**α-T-3'-COOH** (tocopherol derived)								
					PC-3 cells HTB-82 cells 1 µM inhibition (Galli et al., 2004)			m_C6 cells 10 µM reduction (Mazzini et al., 2009)
**γ-T-3'-COOH** (tocopherol derived)								
					PC-3 cells HTB-82 cells 1 µM inhibition (Galli et al., 2004)			m_C6 cells 10 µM reduction (Mazzini et al., 2009)
**δ-T3-13′-COOH**								
PARP-1 CL HCT-116 20 µM induction (Jang et al., 2016)		Casp9 CL HCT-116 20 µM induction (Jang et al., 2016)					m_C6 cells 10 µM reduction (Mazzini et al., 2009)	HCT-116 HT-29 16/17 µM reduction (Jang et al., 2016)
**SCA E**								
PARP-1 CL h_HL-60 25 µM induction (Heo et al., 2011)		Casp9 CL h_HL-60 25 µM induction (Heo et al., 2011)	Casp3 CL h_HL-60 25 µM induction (Heo et al., 2011)		h_HL-60 50 µM inhibition (Heo et al., 2011)			
**α-AC**								
								HepaRG 10 µM no reduction (Richomme et al., 2017)

The effects of the respective compounds on carcinogenesis have been divided into apoptosis-mediated (PARP-1, caspases 3, 7, 8, and 9) activities as well as activities affecting proliferation and viability. The content of each cell of the table in the apoptosis section is constructed as follows (read from top to bottom): (i) investigated parameter; (ii) cell type model used for investigation; (iii) used concentration of the respective compound; (iv) observed effect on the studied parameter; (v) reference. The content of each table cell in the proliferation as well as viability section is constructed as follows (read from top to bottom): (i) cell type model used for investigation; (ii) used concentration of the respective compound; (iii) observed effect on the studied parameter; (iv) reference. The following abbreviations are used: A, activity; A549, adenocarcinomic human alveolar basal epithelial cells; BALB/c mice, albino laboratory-bred strain of the house mouse; LNCap, androgen-sensitive human prostate adenocarcinoma cells; Casp, caspase; MCF-7-C3, caspase 3 reconstituted MCF-7 cells; CL, cleavage; cc, colon cancer; MDA-MB-23, epithelial human breast cancer cell line; NB2A, fast-growing mouse neuroblastoma cell line; h, human; MCF-7, human breast cancer cell line established by Michigan Cancer Foundation-7; SKBR3, human breast cancer cell line isolated by the Memorial Sloan–Kettering Cancer Centere; BT-474, human breast carcinoma ductal cell line; SaOs-2, human cell line derived from primary osteosarcoma; SW-480, human cell line established from a lymph node metastasis; HCT-116, human colon cancer cell line; HT-29, human colorectal adenocarcinoma cell line; CaCo-2, human epithelial colorectal adenocarcinoma cells; THP-1, human immortalized monocyte-like cell line; HL-60, human leukemia cell line; HepG2, human liver cancer cell line; MiaPaCa-2, human pancreatic cancer cell line; Du-145, human prostate cancer cell line; PC-3, human prostate cancer cell line; B16(10), mouse skin melanoma cells; m, murine; m_C6, murine glial cancer cell line; PARP-1, poly (ADP-Ribose)-polymerase 1; HTB-82, rhabdomyosarcoma cell line; rh_RLh-84, rat hepatoma cell line; HepaRG, terminally differentiated hepatic cells derived from a human hepatic progenitor cell line.

All results obtained from in vivo studies are marked in gray.

##### Tocopherols and Tocotrienols

Like the mediation of anti-inflammatory effects, anti-carcinogenic actions were profoundly investigated for α-TOH and γ-TOH, due to their biological relevance. Screening of multiple human breast cancer cell lines (Nesaretnam et al., 1995; Guthrie et al., 1997; Nesaretnam et al., 1998; Birringer et al., 2003; Loganathan et al., 2013) and the human osteosarcoma cell line Saos-2 (Azzi et al., 1993; Gysin et al., 2002) revealed no anti-proliferative effects or alteration of cell viability using α-TOH (4.6–230 µM), whereas Campbell et al. found controversial results for different human colon cancer cell lines using different assays (Campbell et al., 2006). However, the colon cancer cell lines HT-29 (Campbell et al., 2006) and CaCo-2 (Gysin et al., 2002) treated with 100 µM (48 h), 200 µM (5 h), and 25 µM (24 h) α-TOH showed significantly induced cell death and dampened proliferation. In addition, proliferation of different human prostate cancer cells was significantly inhibited by α-TOH. In brief, 25–50 µM α-TOH inhibited proliferation of PC-3 cells (41%, 24 h) (Galli et al., 2004), Du-145 cells (50%, 24 h), and LNCaP cells (48%, 48 h) (Gysin et al., 2002). Furthermore, α-TOH (50 µM) significantly inhibited the growth of murine neuroblastoma NB2A cells by 50% (Azzi et al., 1993), and rhabdomyosarcoma HTB-82 cells by 32% (Galli et al., 2004). However, effects on proliferation and viability seemed to be independent from the cleavage and activity of the apoptosis marker PARP-1 and caspases 3, 7, and 8 in breast cancer (23 µM [Loganathan et al., 2013]), pancreas cancer (50 µM [Husain et al., 2011]), and colon cancer cells (100 µM [Campbell et al., 2006]). β-TOH-treatment of cancer cells revealed similar effects compared to α-TOH. While growth of human prostate cancer cells was significantly inhibited by >40% (Gysin et al., 2002), growth of human osteosarcoma cells was marginally inhibited. In neuroblastoma (Azzi et al., 1993) and breast cancer cells (Birringer et al., 2003) β-TOH did not alter cell viability.

γ-Tocopherol is by far the most potent anti-carcinogenic TOH regarding prostate-cancer. Indeed, viability or rather proliferation of prostate-cancer cell lines PC-3 (1 µM [Galli et al., 2004], 50 µM [Jiang et al., 2012]), CaCo-2, Du-145, LNCaP (25 µM [Gysin et al., 2002]), SW480, HCT-116, HCT-15, and HCT-29 (100 µM [Campbell et al., 2006]) was blocked by γ-TOH. More precisely, 100 µM γ-TOH induced apoptosis in SW480 and HCT-116 cells following the cleavage of PARP-1 as well as caspases 3, 7, and 8 (Campbell et al., 2006). Described effects are most likely tumor-specific, finding no or weak alteration of tumor growth on breast cancer cell lines (Birringer et al., 2003) and colon carcinoma cells (Jang et al., 2016). However, in male BALB/c mice γ-TOH (0.1% of diet) suppressed DSS- and AOM-induced tumor multiplicity of macroscopic adenomas and large adenomatous polyps (>2mm^2^) by 60, 85, and 36% (Jiang et al., 2013). Of the tested tumor cell lines, only viability of HCT-116 was inhibited by 50 µM δ-TOH, whereas HT-29 cells, and the breast cancer cell lines MCF-7 and MCF-7-C3 were not affected (Birringer et al., 2003; Jang et al., 2016). Based on the presented data, anti-carcinogenic capacity for different forms of TOHs can be assessed as γ-TOH >> β-TOH > α/δ-TOH.

Despite of the promising results outlined above, it should be noticed that several human trials failed to confirm preventive effects of vitamin E, in particular α-TOH, against cancer. The Alpha-Tocopherol Beta-Carotene (ATBC) Cancer Prevention Study examined whether a daily supplementation of 50 mg α-TOH and/or 20 mg β-carotene could prevent lung cancer in male smokers (Virtamo et al., 2014). However, after five to eight years of supplementation of either α-TOH or β-carotene or the combination of both failed to prevent lung cancer (Virtamo et al., 2014). In addition, other human intervention trails revealed disappointing results, with the Selenium and Vitamin E Cancer Prevention Trial (SELECT) representing a very interesting one. The aim of the SELECT study was to investigate the preventive potential of α-TOH and/or selenium on prostate cancer. In the SELECT trial, healthy men received a daily dose of either 400 IU all-*rac*-α-tocopheryl acetate or 200 μg selenium or a combination of both for an average of 5.5 years (Lippman et al., 2009). Supplementation with both compounds failed to prevent prostate cancer development. Surprisingly, daily supplementation with all-*rac*-α-tocopheryl acetate was slightly, but not significantly, associated with an increased overall risk for prostate cancer (Lippman et al., 2009). Next, in the 7 to 12 years follow-up the subjects who had received a daily dose of 400 IU all-*rac*-α-tocopheryl acetate showed a significantly enhanced risk for prostate cancer (Klein et al., 2011). This result indicates that a dietary supplementation with high doses of this vitamin E derivate could result in an increased risk for cancer.

The T3-rich fraction of palm oil is comprised of all T3 forms (α- [25%], γ- [29%], δ-T3 [14%] relative to the total vitamin E amount) and inhibits the proliferation of the estrogen receptor‐negative human breast cancer cell line MDA-MB-435 with an IC_50_ of 180 μg/ml (Nesaretnam et al., 1995). Based on that finding, single forms of T3s were tested regarding their effects on proliferation and viability of carcinoma cell lines. The α-, γ-, and δ-forms of T3s were found to mediate cancer type specific effectiveness, with breast cancer cell lines being most affected by the treatment with TOHs. Viability and proliferation of MDA-MB-231 (IC_50_ 22.5 µM), MCF-7 (IC_50_ 14.1–26.1 µM), and MDA-MB-435 cells (IC_50_: 211.9 µM) were concentration-dependently affected by α-T3 treatment independent on whether they were responsive to estrogen and estradiol (Guthrie et al., 1997; Nesaretnam et al., 1998; Loganathan et al., 2013). However, whereas cleavage of PARP-1 (Loganathan et al., 2013) has been observed, general involvement of apoptosis has not been described yet (Birringer et al., 2003). Although cleavage of PARP-1 as well as caspases 3 and 8 has been observed in pancreatic MiaPaCa-2 carcinoma cells, 50 µM α-T3 had no effect on cell viability (Husain et al., 2011). In contrast, β-T3 (50 µM) reduced the viability of MiaPaCa-2 cells (Husain et al., 2011). In mice, 200 mg/kg α-T3 did not affect tumor growth of AsPC-1 human pancreatic cancer xenografts (Husain et al., 2011), whereas 110 µM α-T3 suppressed proliferation of murine B16(F10) melanoma cells (He et al., 1997).

Within the group of TOHs and T3s, γ-T3 is the most potent anti-carcinogenic form that affects cell growth of breast, prostate, pancreas, and hepatic cancer cells, likely due to a preferred incorporation of γ-T3 in these cells (Sakai et al., 2004). There is strong evidence for the anti-proliferative effects of γ-T3 on breast cancer cell lines MDA-MB-231 (IC_50_ 11.4 µM), MCF-7 (IC_50_ 15.4 µM) (Loganathan et al., 2013), SKBR3 (IC_50_ 4 µM), BT474 (IC_50_ 4 µM) (Alawin et al., 2016), estrogen receptor-negative MDA-MB (IC_50_ 73.2 µM), and estrogen receptor-positive MCF-7 cells (IC_50_ 4.9 µM) (Guthrie et al., 1997). Others even found complete inhibition of MCF-7 cell growth by γ-T3 at a concentration of 14.6 µM (Nesaretnam et al., 1998). Inhibitory effects on proliferation were at least in part mediated *via* the activation of apoptosis, such as activation of caspase 3 in MCF-7 (25%), and MCF-7-C3 cells (35%) with 50 µM γ-T3 (Birringer et al., 2003). Furthermore, the proliferation of MiaPaCa-2 pancreas cancer cells (Husain et al., 2011), PC-3 prostate cancer cells (IC_50_: 32 µM, 24 h), and dRLh-84 hepatic cancer cells (IC_50_: 80–100 µM, 24 h) was suppressed by γ-T3, most likely *via* cleavage of PARP-1, and caspases 3, 7, 8, and 9 (Sakai et al., 2004; Yap et al., 2008) and induction of autophagy (Jiang et al., 2012). In murine B16(F10) melanoma cells (He et al., 1997) and the myelogenous leukemia cell line KBM-5 (Ahn et al., 2007) γ-T3 significantly suppressed proliferation (IC_50_ 20 µM, 24 h). Comparable to γ-T3, δ-T3 inhibits the proliferation of the breast cancer cell lines MDA-MB-435 (IC_50_ 226.8 µM), MDA-MB-231 (IC_50_ 17.4 µM), and MCF-7 cells (IC_50_ 5–25.2 µM) (Guthrie et al., 1997; Nesaretnam et al., 1998; Loganathan et al., 2013), as well as prostate cancer cell lines PC-3 (IC_50_ 41 µM), and LNCaP (IC_50_ 75 µM) (Yap et al., 2008), melanoma B16(F10) cells (IC_50_ 10 µM) (He et al., 1997), and MiaPaCa-2 pancreas cancer cells (IC_50_ 50 µM) (Husain et al., 2011) by the induction of apoptosis, as indicated by the cleavage of apoptosis-mediating PARP-1 as well as caspases 3 and 8 (Husain et al., 2011).

##### Metabolites of Tocopherols and Tocotrienols

In contrast to the TOH and T3 forms, the respective metabolites have been rarely investigated regarding their anti-carcinogenic properties. The LCMs of TOHs, namely α-T-13′-COOH (20 µM) and δ-T-13′-COOH (20 µM) induced apoptosis *via* the mitochondrial pathway, which was shown by cleavage of PARP-1 and caspases 3, 7, and 9, resulting in decreased viability of HepG2 cells (IC_50_ 13.5 µM and 6.5 µM, respectively, Birringer et al., 2010). In human leukemia-derived THP-1 macrophages, viability was decreased by α-T-13′-COOH (IC_50_ 7.4 µM, Wallert et al., 2014a) and δ-T-13′-COOH (IC_50_ 11.1 µM, Schmölz et al., 2017). In addition, δ-T-13′-COOH increased apoptosis-induced cytotoxicity in HCT-116 (IC_50_ 8.9 µM), HT-29 (IC_50_ 8.6 µM) (Jang et al., 2016), and C6 cells (IC_50_ <10 µM, Mazzini et al., 2009). The T3-derived δ-garcinoic acid decreased the viability of HCT-116, HT-29 (Jang et al., 2016), glioma C6 (Mazzini et al., 2009), and human THP-1 macrophage-like cells (IC_50_ <20 µM, unpublished data) to a similar extent. In BALB/c mice fed with 0.022%, δ-garcinoic acid in the diet, AOM- and DSS-induced colon tumor growth was decreased (Jang et al., 2016). In contrast to the carboxychromanol structures, the hydroxychromanols were less efficient in the cleavage of apoptosis markers and consequently did not affect the viability of HepG2 cells (Birringer et al., 2010) and THP-1 macrophages (Wallert et al., 2014a; Schmölz et al., 2017) at concentrations up to 50 µM and 100 µM, respectively, whereas an anti-proliferative effect on glioma C6 cancer cells was determined using 10 µM α-T-13′-OH (Mazzini et al., 2009). Short-chain metabolites were found to affect growth of prostate cancer cells PC-3 and rhabdomyosarcoma HTB-82 cells at a concentration of 1 µM (Galli et al., 2004).

##### Sargachromanols

The group of sargachromanols may serve as anti-carcinogenic agents that suppress cell proliferation as reported for SCA E in HL-60 leukemia cells accompanied by cleavage of PARP-1 as well as caspases 3 and 9 (Heo et al., 2011). However, confirmatory data are pending.

##### Amplexichromanols

To date, α-AC has been studied only in HepaRG cells, without effects on viability up to concentrations of 10 µM (Richomme et al., 2017). Therefore, studies on anti-carcinogenic effects of amplexichromanols are still on demand.

#### Chromenols

Within the group of chromenols, δ-sargachromenol is the best-studied one. Previous studies revealed an induction of the cleavage of PARP-1 and caspases along with the induction of apoptosis and reduced cell viability in human skin keratinocyte (HaCaT) cells (Hur et al., 2008). Data obtained from cancer cell lines is still lacking.

## Interference with Molecular Targets and Key Proteins Connecting Inflammation and Carcinogenesis

Many signaling molecules involved in inflammatory processes play in parallel also key roles in carcinogenesis. We here exemplarily focus on the interaction of selected chromanols and chromenols with the molecular crosstalk of NF-κB (Jurjus et al., 2016), lipoxygenases (Rådmark et al., 2015; Roos et al., 2016; Merchant et al., 2018), MAPK (Gkouveris and Nikitakis, 2017; Jiménez-Martínez et al., 2019), and the inflammasome (Moossavi et al., 2018; Swanson et al., 2019) due to their accepted involvement in both, inflammation and cancer (**Figure 7**). However, due to the sparse knowledge about their connection to chromanols and chromenols, further topics, like the interaction of tumor and immune cells, adhesion proteins, structure and regulation of tumor microenvironments, mechanisms for programed cell death as well as other prominent signaling pathways (PI3K/Akt/mTOR; PKC; STAT; Wnt/β-catenin), were not considered in this review.

### Chromanols

A detailed overview on the interference of chromanols with molecular targets and key enzymes connecting inflammation and carcinogenesis is provided in **Table 4**.

**Table 4:** Overview on the interference of chromanols with molecular targets and key enzymes connecting inflammation and carcinogenesis.

NF-кB				NLRP3	MAPKs	Lipoxygenases		
**α-TOH**								
PMA NF-кB A BALB c/3T3 fibroblasts 50 µM no inhibition (Azzi et al., 1993)	LPS NF-кB PE (Nucleus) RAW264.7 100 µM induction (Wallert et al., 2015)	IL-1β, IL-6, TNF-α, LPS, PGE_2_, INF-γ h_DC NF-кB A 500 µM inhibition (Tan et al., 2005)	IL-1β, IL-6, TNF-α, LPS, PGE_2_, INF-γ h_DC IкB-α Phos 500 µM inhibition (Tan et al., 2005)			– 5-LO A enzyme 5 µM inhibition (Reddanna et al., 1985)	– 5-LO A enzyme >1 µM inhibition (Pein et al., 2018)	AA 5-LO PF PMNL 808 µM inhibition (Pein et al., 2018)
– p65 DNA BA MiaPaCa-2 50 µM no inhibition (Husain et al., 2011)						AA 12-LO PF PMNL 3 µM no inhibition (Pein et al., 2018)	AA 15-LO PF PMNL 3 µM no inhibition (Pein et al., 2018)	
**β-TOH**								
PMA NF-кB A Balb c/3T3 fibroblasts 50 µM no inhibition (Azzi et al., 1993)						– 5-LO A enzyme 750 nM inhibition (Pein et al., 2018)	AA 12-LO PF PMNL 3 µM no inhibition (Pein et al., 2018)	AA 15-LO PF PMNL 3 µM no inhibition (Pein et al., 2018)
						AA 5-LO PF PMNL 57 µM inhibition (Pein et al., 2018)		
**γ-TOH**								
TNF-α NF-кB Actv KBM-5 25 µM no inhibition (Ahn et al., 2007)						– 5-LO A enzyme 2–3 µM inhibition (Reddanna et al., 1985)	AA 5-LO PF PMNL 502 µM inhibition (Pein et al., 2018)	– 5-LO A enzyme 910 nM inhibition (Pein et al., 2018)
						– 5-LO A enzyme > 50 µM no inhibition (Jang et al., 2016)	AA 12-LO PF PMNL 3 µM no inhibition (Pein et al., 2018)	AA 15-LO PF PMNL 3 µM no inhibition (Pein et al., 2018)
						– 5-LO A enzyme 200 µM inhibition (Jiang et al., 2011)		
**δ-TOH**								
						– 5-LO A enzyme 310 nM inhibition (Pein et al., 2018)	– 5-LO A enzyme > 50 µM no inhibition (Jang et al., 2016)	
						AA 5-LO PF PMNL 85 µM inhibition (Pein et al., 2018)	AA 12-LO PF PMNL 3 µM no inhibition (Pein et al., 2018)	AA 15-LO PF PMNL 3 µM inhibition (Pein et al., 2018)
**α-T3**								
– p65 DNA BA MiaPaCa-2 50 µM no inhibition (Husain et al., 2011)	– p65 Trl MiaPaCa-2 50 µM no inhibition (Husain et al., 2011)	– p65 Trl AsPC-1 50 µM no inhibition (Husain et al., 2011)	– p65 Trl m_TT 50 µM no inhibition (Husain et al., 2011)			– 5-LO A enzyme 330 nM inhibition (Pein et al., 2018)	AA 12-LO PF PMNL 3 µM inhibition (Pein et al., 2018)	AA 15-LO PF PMNL 3 µM no inhibition (Pein et al., 2018)
						AA 5-LO PF PMNL 277 µM inhibition (Pein et al., 2018)		
**β-T3**								
– p65 DNA BA (Cytosol) MiaPaCa-2 50 µM inhibition (Husain et al., 2011)	– p65 DNA BA (Nucleus) MiaPaCa-2 50 µM no inhibition (Husain et al., 2011)	– p65 Trl MiaPaCa-2 50 µM inhibition (Husain et al., 2011)	– p65 Trl m_TT 50 µM no inhibition (Husain et al., 2011)			– 5-LO A enzyme 190 nM inhibition (Pein et al., 2018)	AA 12-LO PF PMNL 3 µM inhibition (Pein et al., 2018)	AA 15-LO PF PMNL 3 µM no inhibition (Pein et al., 2018)
– p65 Trl AsPC-1 50 µM no inhibition (Husain et al., 2011)						AA 5-LO PF PMNL 95 µM inhibition (Pein et al., 2018)		
**γ-T3**								
– p65 Trl PC3 cells 40 µM inhibition (Yap et al., 2008)	TNF-α NF-кB Actv RAW264.7 20 µM inhibition (Wang et al., 2015)	TNF-α NF-кB Actv H1299, A293, MCF-7, U226, SCC4 25 µM inhibition (Ahn et al., 2007)	diabetes IкB-α PE *db/db* mice 0.1% of diet induction (Kim et al., 2016)	LPS/pal, Ng NLRP3 E m_BMDM 1 µM inhibition (Kim et al., 2016)	diabetes p38 Phos db/db mice 0.1% of diet inhibition (Kim et al., 2016)	- 5-LO A enzyme 200 nM inhibition (Pein et al., 2018)	AA 12-LO PF PMNL 3 µM inhibition (Pein et al., 2018)	AA 15-LO PF PMNL 3 µM no inhibition (Pein et al., 2018)
– NF-кB Actv mice 400 mg/kg/d inhibition (Kunnumakkara et al., 2010)	– p65 DNA BA MiaPaCa-2 50 µM inhibition (Husain et al., 2011)	– p65 Trl AsPC-1 50 µM inhibition (Husain et al., 2011)	TNF-α IкB-α PE RAW264.7, m_BMDM 20 µM induction (Wang et al., 2015)	diabetes NLRP3 E db/db mice derived PM/AT 0.1% of diet inhibition (Kim et al., 2016)	diabetes ERK Phos db/db mice 0.1% of diet inhibition (Kim et al., 2016)	AA 5-LO PF PMNL 132 µM inhibition (Pein et al., 2018)	LPS/pal 5-LO GE BMDM 1 µM no inhibition (Kim et al., 2018)	
– p65 Trl MiaPaCa-2 50 µM no inhibition (Husain et al., 2011)	– p65 Trl m_TT 50 µM inhibition (Husain et al., 2011)	TNF-α IкB-α Phos A549, PC3, MCF-7 20 µM inhibition (Wang et al., 2015)	LPS/pal IкB-α PE m_BMDM 1 µM induction (Kim et al., 2018)		TNF-α JNK Phos RAW264.7, m_BMDM 20 µM inhibition (Wang et al., 2015)			
– IкB-α Phos AsPC-1 50 µM inhibition (Husain et al., 2011)	– IкB-α Phos MiaPaCa-2 50 µM inhibition (Husain et al., 2011)	– IкB-α Phos m_TT 50 µM inhibition (Husain et al., 2011)			LPS ERK Phos m_BMDM 0.5 µM inhibition (Kim et al., 2016)			
**δ-T3**								
– p65 DNA BA MiaPaCa-2 50 µM inhibition (Husain et al., 2011)	– p65 Trl AsPC-1 50 µM inhibition (Husain et al., 2011)	– p65 Trl MiaPaCa-1 50 µM inhibition (Husain et al., 2011)	– IкB-α Phos MiaPaCa-2, 50 µM inhibition (Husain et al., 2011)			– 5-LO A enzyme 170 nM inhibition (Pein et al., 2018)	AA 12-LO PF PMNL 3 µM inhibition (Pein et al., 2018)	AA 15-LO PF PMNL 3 µM no inhibition (Pein et al., 2018)
– p65 Trl m_TT 50 µM inhibition (Husain et al., 2011)	– IкB-α Phos MiaPaCa-2, 50 µM inhibition (Husain et al., 2011)	– IкB-α Phos m_TT 50 µM inhibition (Husain et al., 2011)				AA 5-LO PF PMNL 60 µM inhibition (Pein et al., 2018)		
**α-T-13′-OH**								
						– 5-LO A enzyme 350 nM inhibition (Pein et al., 2018)	AA 12-LO PF PMNL 3 µM inhibition (Pein et al., 2018)	AA 15-LO PF PMNL 3 µM induction (Pein et al., 2018)
						AA 5-LO PF PMNL 190 nM inhibition (Pein et al., 2018)		
**α-T-13′-COOH**								
LPS p65 Trl RAW264.7 2.5 µM no inhibition (Wallert et al., 2015)						– 5-LO A enzyme 270 nM inhibition (Pein et al., 2018)	AA 12-LO PF PMNL 3 µM inhibition (Pein et al., 2018)	AA 15-LO PF PMNL 3 µM inhibition (Pein et al., 2018)
						AA 5-LO PF PMNL 80 nM inhibition (Pein et al., 2018)		
**δ-T-13′-OH**								
						– 5-LO A enzyme 120 nM inhibition (Pein et al., 2018)	AA 12-LO PF PMNL 3 µM inhibition (Pein et al., 2018)	AA 15-LO PF PMNL 3 µM induction (Pein et al., 2018)
						AA 5-LO PF PMNL 540 nM inhibition (Pein et al., 2018)		
**δ-T-13′-COOH**								
						– 5-LO A enzyme >1 µM inhibition (Pein et al., 2018)	AA 12-LO PF PMNL 3 µM inhibition (Pein et al., 2018)	AA 15-LO PF PMNL 3 µM induction (Pein et al., 2018)
						AA 5-LO PF PMNL 2 µM inhibition (Pein et al., 2018)	– 5-LO A enzyme 2 µM inhibition (Jang et al., 2016)	Ca^2+^ 5-LO PF HL-60 50 µM inhibition (Jiang et al., 2011)
						– 5-LO A enzyme 0.5–1 µM inhibition (Jiang et al., 2011)		
**α-T-5′-COOH**								
						– 5-LO A enzyme 750 nM inhibition (Pein et al., 2018)	AA 12-LO PF PMNL 3 µM inhibition (Pein et al., 2018)	AA 15-LO PF PMNL 3 µM no inhibition (Pein et al., 2018)
**α-T-3′-COOH**								
						– 5-LO A enzyme >3 µM inhibition (Pein et al., 2018)	AA 12-LO PF PMNL 3 µM inhibition (Pein et al., 2018)	AA 15-LO PF PMNL 3 µM no inhibition (Pein et al., 2018)
**δ-T3-13′-COOH**								
						– 5-LO A enzyme 35 nM inhibition (Pein et al., 2018)	AA 12-LO PF PMNL > 3 µM no inhibition (Pein et al., 2018)	AA 15-LO PF PMNL > 3 µM no inhibition (Pein et al., 2018)
						AA 5-LO PF PMNL 260 nM inhibition (Pein et al., 2018)	– 5-LO A enzyme 57 nM inhibition (Richomme et al., 2017)	AA 5-LO PF PMNL 345 nM inhibition (Richomme et al., 2017)
						– 5-LO A enzyme 1 µM inhibition (Jang et al., 2016)		
**SCA D**								
LPS p65 Phos RAW264.7 60 µM inhibition (Heo et al., 2014)	LPS IкB-α Phos RAW264.7 60 µM inhibition (Heo et al., 2014)				LPS JNK Phos RAW264.7 30 µM inhibition (Heo et al., 2014)			
					LPS ERK Phos RAW264.7 30 µM inhibition (Heo et al., 2014)			
**SCA E**								
					LPS ERK Phos RAW264.7 58 µM inhibition (Lee et al., 2013)			
					LPS p38 Phos RAW264.7 58 µM inhibition (Lee et al., 2013)			
					LPS JNK Phos RAW264.7 58 µM inhibition (Lee et al., 2013)			
**SCA G**								
IL-1β p65/p50 Phos MG-63 40 µM inhibition (Yoon et al., 2012b)			IL-1β IкB-α Phos MG-63 20 µM inhibition (Yoon et al., 2012b)		IL-1β ERK Phos MG-63 40 µM inhibition (Yoon et al., 2012b)			
					IL-1β p38 Phos MG-63 20 µM inhibition (Yoon et al., 2012b)			
					IL-1β JNK Phos MG-63 40 µM inhibition (Yoon et al., 2012b)			

The content of each cell of the table is constructed as follows (read from top to bottom): (i) used stimulus; (ii) investigated parameter; (iii) cell type, tissue, mouse, or other models used for investigation; (iv) used concentration of the respective compound; (v) observed effect on the studied parameter; (vi) reference. In the publications where no stimulus was used or was required for the studies, the respective row is marked with “-”. Actv, activation; A, activity; AT, adipose tissue; BALB/c mice, albino laboratory-bred strain of the house mouse; AA, arachidonic acid; BA, binding affinity; BMDM, bone marrow derived macrophages; JNK, c-Jun N-terminal kinase; DC, dendritic cells; E, expression; ERK, extracellular-signal regulated kinase; 3T3, murine fibroblast cell line; GE, gene expression; h, human; MCF-7, human breast cancer cell line established by Michigan Cancer Foundation-7; SCC4, human head and neck squamous cell carcinoma cell line; HL-60, human leukemia cell line; H1299, human non-small cell lung carcinoma cell line; MiaPaCa-2, human pancreatic cancer cell line; AsPC-1, human pancreas adenocarcinoma cell line; U226, human peripheral blood myeloma, plasmacytoma cell line; MG-63, human osteosarcoma cell line; INF-γ, interferon γ; IL, interleukin; db/db mice, leptin receptor activity deficient mice; LPS, lipopolysaccharide; LO, lipoxygenase; RAW264.7, macrophages derived from abelson murine leukemia virus-induced tumor; MAPK, mitogen-activated protein kinase; m, murine; KBM-5, murine myelogenous leukemia cell line; Ng, nigericine; NLRP3, NLR family pyrin domain containing 3; NF-кB, nuclear factor kappa-light-chain-enhancer of activated B cells; p65, nuclear factor NF-кB p65 subunit; IкB, nuclear factor of kappa light polypeptide gene enhancer in B-cells inhibitor; pal, palmitate; PM, peritoneal macrophages; PMA, phorbol-12-myristat-13-acetat; Phos, phosphorylation; PMNL, polymorphonuclear neutrophils; PF, product formation; PGE_2_, prostaglandin E_2_; PE, protein expression; Trl, translocation; TNF-α, tumor necrosis factor α; TT, tumor tissue.

All results obtained from in vivo studies are marked in gray.

#### Tocopherols and Tocotrienols

As outlined above, inflammation and carcinogenesis are only marginally affected by α-TOH. This is probably the consequence of a lack of interference of α-TOH with NF-κB. Neither in phorbol-12-myristat-13-acetate (PMA)-stimulated BALBc/3T3 fibroblasts (Azzi et al., 1993), and human pancreatic cancer MiaPaCa-2 cells (Husain et al., 2011), nor TNF-α-stimulated murine myelogenous leukemia KBM-5 cells (Ahn et al., 2007), α-TOH (50 µM), β-TOH (50 µM), or γ-TOH (25 µM) affected NF-κB binding affinity or its activation. In murine RAW264.7 macrophages, 100 µM α-TOH even induced translocation of p65 into the nucleus (Wallert et al., 2015). However, pharmacological doses of α-TOH (500 µM) inhibited NF-κB transcriptional activity as well as the phosphorylation and subsequent degradation of nuclear factor of kappa light polypeptide gene enhancer in B-cells inhibitor (IκB)-α, the inhibitor of NF-κB, resulting in decreased NF-κB activation in multifactorially stimulated dendritic cells (Tan et al., 2005). γ-Tocotrienol and δ-T3 significantly decreased NF-κB/p65 binding affinity in MiaPaCa-2 cells and diminished p65 subunit translocation in AsPc-1 cells and tumor tissue. In addition, β-T3 and δ-T3 inhibited the translocation in MiaPaCa-2 cells (Husain et al., 2011). The NF-κB inhibitor IκB-α remained unchanged in the aforementioned study. Within the group of T3s, γ-T3 has been described to affect NF-κB activation and p65 subunit translocation in various cell lines and isolated tissue. For example, γ-T3 (20–40 µM) inhibited the phosphorylation of IκB-α and the nuclear translocation of the p65 subunit following various stimuli, including pro-inflammatory cytokines, tumor promoters, carcinogens, and growth factors in different cell lines (Ahn et al., 2007; Yap et al., 2008; Wang et al., 2015). Further, γ-T3 treatment also increased IκB-α protein expression in epididymal adipose tissues isolated from γ-T3-fed *db/db* mice (Kim et al., 2016) as well as in LPS/palmitate-activated BMDM using 1 µM γ-T3 (Kim et al., 2018). In mice, 400 mg γ-T3/kg, applied orally, sensitized pancreatic tumors to gemcitabine treatment, a drug applied in clinical treatment of pancreatic cancer, by suppressing NF-κB-mediated inflammatory pathways linked to tumorigenesis (Kunnumakkara et al., 2010). The expression of A20 (acronym: TNFAIP3), another inhibitor of NF-κB, was induced by 20 µM γ-T3 in RAW264.7, A549, PC3, and MCF-7 cells (Wang et al., 2015) as well as in peritoneal macrophages obtained from diabetic *db/db* mice fed with a γ-T3-containing diet (0.1%) (Kim et al., 2016).

5-, 12-, and 15-LO pathways mediate the formation of lipid mediators (including leukotrienes, lipoxins, resolvins, protectins, and maresins), which orchestrate inflammation by triggering immune cell recruitment and allergic responses, and/or actively terminating inflammation, i.e. triggering resolution of inflammation. Leukotrienes and the so-called specialized pro-resolving lipid mediators (produced by the tumor-microenvironment, in particular by 15-LO-expressing macrophages of the M2 subtype) have further been shown to play pivotal roles in tumor initiation and development as well as angiogenesis and metastasis (Serhan, 2014; Rådmark et al., 2015; Wculek and Malanchi, 2015; Gilligan et al., 2019). All forms of TOHs inhibit the activity of the isolated 5-LO enzyme in the following sequence of their inhibitory capacity: δ-TOH (IC_50_ 0.31 µM) < β-TOH (IC_50_ 0.75 µM) < α-TOH (IC_50_ 1–5 µM) = γ-TOH (IC_50_ 0.9–3 µM) (Reddanna et al., 1985; Pein et al., 2018). In activated polymorphonuclear leukocytes (PMNL), inhibitory concentrations are 10- to100-fold higher with the following order: β-TOH < δ-TOH < γ-TOH < α-TOH (Pein et al., 2018). However, activity of 12- and 15-LO, which catalyze the formation of 12- and 15-HETE, respectively, remained unaltered by 3 µM TOH in LPS-activated PMNL, except for δ-TOH which inhibited 15-LO with an IC_50_ of 3 µM (Pein et al., 2018). α-, β-, γ-, and δ-T3 appeared as efficient inhibitors of isolated 5-LO, all with IC_50_ values below 0.5 µM, whereas the inhibition of 5-LO product formation in activated PMNL required concentrations of 60 µM (δ-T3) to 277 µM (α-T3) (Pein et al., 2018). 12-Lipoxygenase product formation in PMNL was significantly inhibited by all T3 forms, whereas 15-LO-derived products remained unchanged or were even significantly elevated using concentrations of 3 µM (Pein et al., 2018).

MAPK pathways mediate a multitude of cellular processes, including growth, proliferation, differentiation, migration, apoptosis, and inflammation, in response to external stress signals. Therefore, MAPK pathways represent interesting targets for the development of anti-carcinogenic as well as anti-inflammatory therapeutics. Within the MAPK protein family, extracellular signal-regulated kinase (ERK) represents a prominent target for cancer research, because ERK deregulation is linked to approximately one-third of all human cancers (Dhillon et al., 2007). In addition, ERK affects cellular inflammation *via* modulation of cytokine expression (Kim, 2014). However, the stress-activated kinases, c-Jun N-terminal kinase (JNK) and p38, have emerged as interesting therapeutic targets, due to their involvement in the regulation of inflammation, DNA damage response, and apoptosis (Kaminska, 2005). Inhibitory effects of γ-T3 on the MAPK pathway, more precisely the phosphorylation of ERK, p38 and JNK have been observed in epididymal adipose tissues from γ-T3-fed *db/db* mice (0.1% of the diet), in LPS-activated BMDMs using 0.5 µM γ-T3 (Kim et al., 2016), and in TNF-α-activated RAW264.7 cells (Wang et al., 2015). The relevance of NLRP3 inflammasome activation and subsequent formation of pro-inflammatory cytokines, namely IL-1β and IL-8, in inflammation and related diseases has been shown. γ-T3 decreased NLRP3 inflammasome activation by inhibiting the mRNA and protein expression of the NLRP3 inflammasome in BMDM activated with LPS/palmitate, rather than with LPS/nigericin, in peritoneal macrophages and adipose tissue isolated from γ-T3-fed *db/db* mice (Kim et al., 2016). In addition, in BMDMs treated with chloroquine, an inhibitor of lysosomal degradation, the accumulation of microtubule-associated protein 1A/1B-light chain 3 (LC3)-II, and the degradation of p62 were decreased implying that γ-T3 co-regulates autophagosome formation and inflammasome activation (Kim et al., 2016).

#### Metabolites of Tocopherols and Tocotrienols

Tocopherols and T3s inhibit the activity of isolated recombinant human 5-LO enzyme 10- to 100-fold more efficiently than in activated PMNL. The respective long-chain TOH- and T3-derived metabolites inhibited isolated 5-LO to a similar extent (Jiang et al., 2011; Jang et al., 2016; Pein et al., 2018). Notably, in activated PMNL, α-T-13′-COOH was the most potent inhibitor of 5-LO activity with an IC_50_ value of 80 nM followed by α-T-13′-OH (190 nM), δ-T-13′-OH (540 nM), and δ-T-13′-COOH (2 µM) (Pein et al., 2018). Treatment of activated PMNL with 3 µM LCM effectively blocked 12- and 15-LO product formation, whereas only the 12-LO pathway was blocked by α-5′-T-COOH, α-3′-T-COOH, and γ-3′-T-COOH (Pein et al., 2018). Conversion of arachidonic acid to leukotrienes *via* 5-LO was blocked by δ-T3-13′-COOH (human recombinant enzyme: IC_50_ 35–57 nM) (Richomme et al., 2017; Pein et al., 2018) and 1 µM (Jang et al., 2016); neutrophils (IC 260–345 nM) (Richomme et al., 2017; Pein et al., 2018), whereas product formation mediated by 12- and 15-LO remained unchanged (Jang et al., 2016). The discrepancy in IC_50_ values in the inhibition of cell-free 5-LO likely depends on the different assay conditions. While Pein et al. analyzed specific 5-LO products by reverse-phase high-performance liquid chromatography with ultraviolet detection, Jang et al. used an indirect colorimetric assay, which determines the formation of hydroperoxides. For SCMs, namely 5′-T-COOH and 3′-T-COOH, no inhibitory effect was observed at the tested concentrations up to 3 µM, except for α-5′-T-COOH (IC_50_ 750 nM) (Pein et al., 2018).

#### Sargachromanols

Blocking of NF-κB activation with SCAs by inhibiting the phosphorylation of p65 and IκB-α, thereby protecting IκB-α from degradation, has been shown in LPS-activated RAW264.7 macrophages (Heo et al., 2014) and in IL-1β-activated MG-63 osteosarcoma fibroblasts (Yoon et al., 2012b) for SCA D (60 µM) and G (20 µM), respectively. In addition, interference of SCAs D, E, and G with the MAPK pathways, namely phosphorylation of JNK, ERK, and p38, has been observed in LPS-stimulated RAW264.7 macrophages and IL-1β-activated MG-63 osteosarcoma fibroblasts (Yoon et al., 2012b; Lee et al., 2013; Heo et al., 2014).

### Chromenols

Like SCAs, δ-SCE has been shown to interfere with the NF-κB and the MAPK pathways. In TNF-α-stimulated endothelial cells (Gwon et al., 2017) and LPS-stimulated microglia cells (Kim et al., 2018), p65 translocation and the phosphorylation of IκB-α were inhibited by 40 µM and 60 µM δ-SCE, respectively. In the same cell models inflammation-induced phosphorylation of JNK and ERK was diminished by δ-SCE, whereas p38 remained unchanged (Kim et al., 2018) (**Table 5**).

**Table 5:** Overview on the interference of chromenols with molecular targets and key enzymes connecting inflammation and carcinogenesis.

NF-кB				MAPKs		
**Sargachromenol**						
TNF-α p65 Trl HUVEC 40 µM inhibition (Gwon et al., 2017)	TNF-α p65 PE HUVEC 40 µM inhibition (Gwon et al., 2017)	LPS p65 Trl BV-2 60 µM inhibition (Kim et al., 2014)	TNF-α IкB-α Phos HUVEC 40 µM inhibition (Gwon et al., 2017)	LPS JNK Phos BV-2 60 µM inhibition (Kim et al., 2014)	LPS p38 Phos BV-2 60 µM no inhibition (Kim et al., 2014)	LPS ERK Phos BV-2 60 µM inhibition (Kim et al., 2014)
TNF-α IкB-α Phos HUVEC 40 µM inhibition (Gwon et al., 2017)	LPS IкB-α Phos BV-2 60 µM inhibition (Kim et al., 2014)					

The content of each cell of the table is constructed as follows (read from top to bottom): (i) used stimulus; (ii) investigated parameter; (iii) cell type tissue, mouse, or other models used for the studies; (iv) used concentration of the respective compound; (v) observed effect on the studied parameter; (vi) reference. The following abbreviations are used. BV-2, brain microglial cells transformed by recombinant retrovirus (v-raf/v-mic); JNK, c-Jun N-terminal kinase; ERK, extracellular-signal regulated kinase; HUVEC, human umbilical vein endothelial cells; LPS, lipopolysaccharide; NF-кB, nuclear factor kappa-light-chain-enhancer of activated B cells; p65, nuclear factor NF-кB p65 subunit; IкB, nuclear factor of kappa light polypeptide gene enhancer in B-cells inhibitor; Phos, phosphorylation; PE, protein expression; Trl, translocation; TNF-α, tumor necrosis factor α.

## Low and High-Affinity Molecular Targets

**Figure 8:**
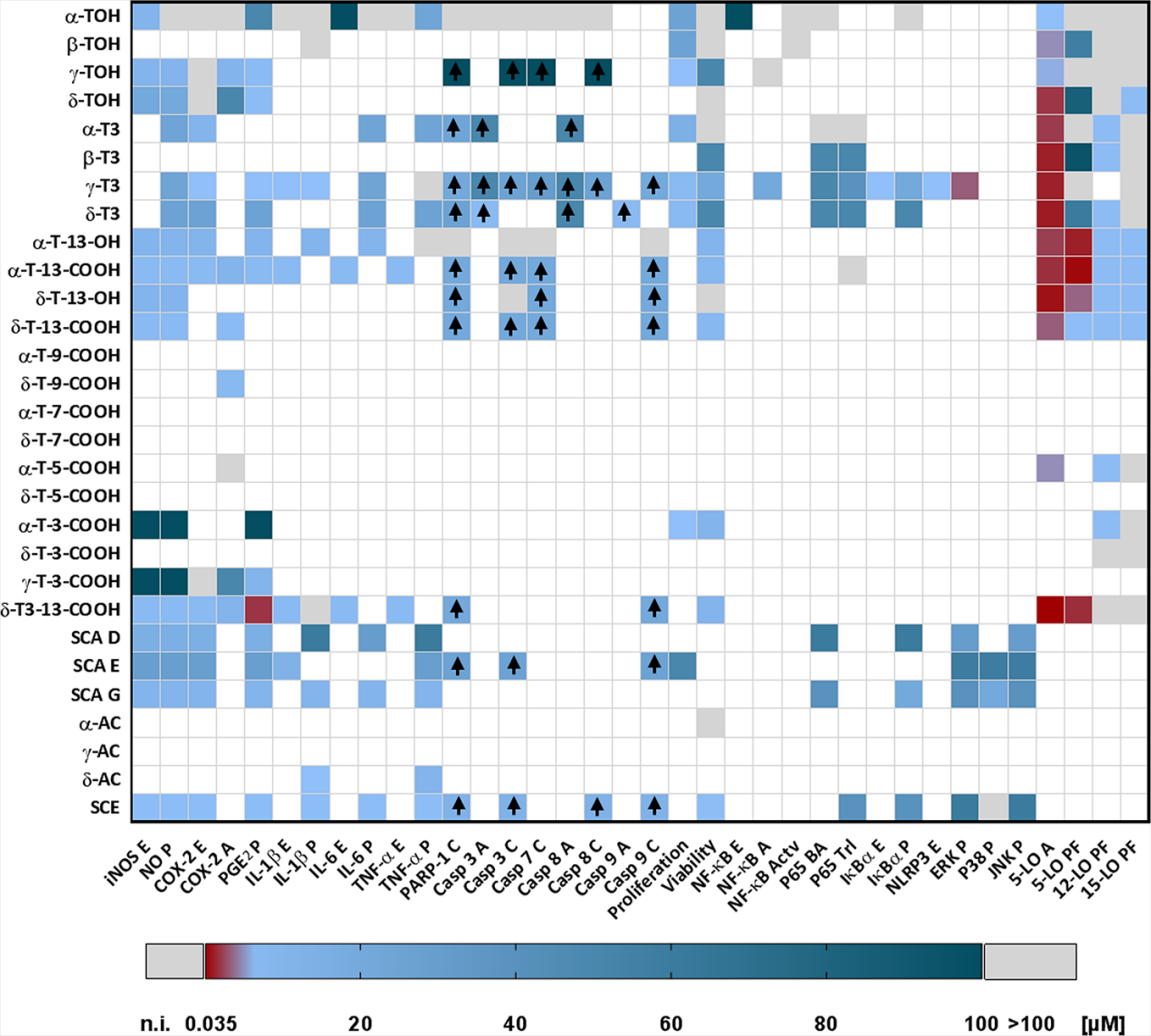
Heatmap illustrating the effectiveness of chromanol and chromenol structures on selected targets. If not indicated otherwise, the plotted effects represent inhibitory effects of the respective compound on distinct parameters; induced parameters are marked with an arrow (↑). The color coding of the presented heat map ranges from high-affinity targets and parameters (effect with <1 µM) presented in red to low-affinity targets and parameters (effects with >1 µM to ≤100 µM) presented in dark blue. If a compound did not affect a specific factor/parameter or showed low effectiveness (>100 µM), the factor/parameter is marked in light gray. Factors and parameters lacking data are marked in white. The heat map is considered as simplified guide for orientation and does not provide a detailed summary of the topic. All concentrations are given micromole (µM). Abbreviations used are: Actv, activation; A, activity; BA, binding affinity; C, cleavage; E, expression; PF, product formation; P, production; T, translocation.

The heat map in **Figure 8** provides a simplified overview about high- and low-dose bioactivities of the different chromanols and chromenols for a rapid assessment. The selection of compounds and parameters is based on a comprehensive review of the current literature about chromanols and chromenols and focusses on the important biological functions described for these compounds in the context of inflammation and cancer. For reasons of simplification, we did not take into account compound-specific uptake kinetics or cell type- or animal model-specific differences. For more detailed information, the reader is referred to **Tables 1** **–** **5** which summarize our current knowledge on the chromanols and chromenols described in the respective sections. For comparison, presented concentrations are IC_50_ values or the lowest reported concentrations affecting the respective parameters.

In the studies considered here, T3s often showed higher effectiveness on the induction or suppression of biological activities linked to inflammation and cancer than TOHs. Furthermore, oxidative modification of the terminal side-chain often substantially increases the anti-inflammatory capacity of respective compounds compared to parental compounds, such as TOHs and T3s. Amplexichromanols, sargachromanols and sargachromenols are also characterized by oxidative modifications of the side-chain, which might rationalize potent interactions with inflammatory targets, which needs further investigation. Notably, regulation of different target genes, proteins, and nuclear receptors can hardly be generalized. For instance, within the group of investigated targets, 5-LO is mostly inhibited by a few compounds, with δ-T3-13′-COOH showing strongest inhibitory effects (IC_50_ 35 nM) and α-TOH showing the least (IC_50_ 1 µM). In contrast, the COX-2-regulated formation of signaling molecules is most efficiently inhibited by γ-T3. In summary, especially 5-LO seems to represent a high affinity (affected at concentrations <1 µM) and therefore specific target for the LCMs of vitamin E. Most of the other observed effects, like mediation of caspase activity, anti-proliferative effects, inhibition of NO formation, are probably the result of a stimulation involving low-affinity targets (affected at concentrations ≥1 µM). However, as implied by the heat map in **Figure 8**, further studies are required for a comprehensive evaluation of the potential of chromanol and chromenol structures to serve as lead structures for the development of future anti-inflammatory therapeutic approaches.

## Conclusion

For our review, we selected chromanols and chromenols for which data on anti-inflammatory and anti-carcinogenic effects were available in public databases of the scientific literature. The structures of our interests were tocopherols, tocotrienols, and their respective metabolites (which are produced in the liver under physiological and pathophysiological conditions) as well as structurally related compounds including sargachromanols, sargachromenols, and amplexichromanols. Criteria for the evaluation of compounds as possible lead structures for future therapeutic targets were their effects on key inflammatory and apoptotic pathways, proliferation, and interaction with (nuclear) receptor and enzymes that connect inflammation with carcinogenesis. Within this group of selected structures, tocopherols, more precisely α-TOH, are by far the most extensively studied compounds. However, the effects of TOHs are mostly only marginal compared to other compounds described in this review.

It should be noted that the methylation pattern of the chromanol ring system significantly affects inflammation and carcinogenesis. For instance, non-α-TOH and non-α-T3 forms affect eicosanoid- and cytokine-mediated inflammation as well as the cleavage of caspases that mediate apoptosis. Further, T3s are more potent in inhibiting caspase cleavage compared to the respective TOH forms. Tocopherol- and T3-derived metabolites and carboxychromanols more than hydroxychromanols inhibit LO, and in particular 5-LO, effectively and reduce the viability of multiple cancer cell lines. Furthermore, sargachromanols interact with MAPK and NF-κB pathways, assuming their crosstalk with both, carcinogenesis and inflammation, while sargachromenols mediate anti-carcinogenic effects. Although our knowledge about biological activities of amplexichromanols is sparse, first results indicate their potential for pharmacological applications.

The development of clinically relevant nitric oxide-, eicosanoid-, or cytokine-inhibiting agents or agents that interact with signaling pathways of inflammation is challenging with respect to selectivity and toxicity. Next, although blocking inflammation is meant to be protective, its permanent or long-term inhibition may cause damage to the body (Brasky et al., 2017). Although detrimental effects of naturally occurring chromanols and chromenols cannot be excluded yet, they are less likely for this group of lead compounds in light of the good tolerability of TOHs and T3s at low to moderate doses. Further studies are required to evaluate whether the observed effects of chromanols and chromenols on inflammation and carcinogenesis are indeed beneficial in humans. Until today, no human clinical trials have been published that provide valid information on the biological activity, bioavailability, kinetics, systemic distribution, or local accumulation of these compounds. However, this groups of molecules appears to be promising as lead structures for future anti-inflammatory and/or anti-cancerogenic therapeutic approaches.

## Limitations

Our review is based on a recent systematic review of Birringer et al. (2018), which presented the first comprehensive overview on the diversity of chromanol and chromenol structures and their biological functions. The aim of our review was to more selectively describe the effects on signaling pathways involved in inflammation, apoptosis, cell proliferation, and carcinogenesis and the underlying modes of action for selected chromanols and chromenols. We are aware of the lack of data for a variety of chromenol structures in our overview. We therefore focused on chromanols and chromenols only where adequate data was available that reported anti-inflammatory and anti-carcinogenic properties. For a more detailed description of the structural and chemical properties of all 230 chromanol and chromenol structures, the reader is referred to (Birringer et al., 2018).

## Author Contributions

MW and SK wrote the manuscript. MW, SK, MS, MB, and SL designed and structured the manuscript, MS, MB, SL, AK, and OW supervised the project and carefully read, evaluated, and discussed the content of the manuscript.

## Funding

SL and OW were supported by the Free State of Thuringia and the European Social Fund (2016 FGR 0045), and The Deutsche Forschungsgemeinschaft (CRC 1278 “Polymer-based nanoparticle libraries for targeted anti-inflammatory strategies (PolyTarget). Work of SL and AK was supported by the Deutsche Forschungsgemeinschaft (DFG, RTG 1715), and AK was funded by the DFG (KO 4589/7-1). In addition, work of OW and SL is also funded within the Collaborative Research Centre (SFB) 1278 (PolyTarget) by the DFG. Other sources of funding include the Forschungskreis der Ernährungsindustrie (FEI) as part of an AiF (Arbeitsgemeinschaft industrieller Forschungsvereinigungen “Otto von Guericke”) project of the Industrielle Gemeinschaftsforschung (IGF), and the German Federal Ministry of Education and Research (nutriCARD, grant agreement number 01EA1411A).

## Conflict of Interest

The authors declare that the research was conducted in the absence of any commercial or financial relationships that could be construed as a potential conflict of interest.

## Abbreviations

α-T-13′-COOH, α-13′-carboxychromanol; α-T-13′-OH, α-13′-hydrochromanol; δ-T3-13′-COOH, garcinoic acid; AC, amplexichromanol; BMDM, bone marrow-derived macrophages; CEHC, carboxyethyl-hydroxychromanol; JNK, c-Jun N-terminal kinase; CoA, coenzyme A; COX, cyclooxygenases; CYP, cytochrome P450; ERK, extracellular signal-regulated kinase; ICM, intermediate-chain metabolite; IL, interleukin; iNOS, inducible nitric oxide synthase; IкB, nuclear factor of kappa light polypeptide gene enhancer in B-cells inhibitor; LCM, long-chain metabolite; LPS, lipopolysaccharide; LO, lipoxygenases; MAPKs, mitogen-activated kinases; NF-кB, nuclear factor kappa-light-chain-enhancer of activated B cells; NLRP3, NLR family pyrin domain containing 3; NO, nitric oxide; PARP-1, poly-[ADP-ribose]-polymerase 1; PG, prostaglandin; PMA, phorbol-12-myristat-13-acetate; PMNL, polymorphonuclear neutrophils; ROS, reactive oxygen species; SCA, sargachromanols; SCE, sargachromenols; SCM, short-chain metabolites; TX, thromboxane; TOH, tocopherol; TNF-α, tumor necrosis factor α; T3, tocotrienol.

## References

[B1] Aggarwal B. B. (2009). Inflammation, a silent killer in cancer is not so silent! Curr. Opin. Pharmacol. 9, 347–350. 10.1016/j.coph.2009.06.018 19671496

[B2] Ahn K. S. Sethi G. Krishnan K. Aggarwal B. B. (2007). Gamma-tocotrienol inhibits nuclear factor-kappaB signaling pathway through inhibition of receptor-interacting protein and TAK1 leading to suppression of antiapoptotic gene products and potentiation of apoptosis. J. Biol. Chem. 282, 809–820. 10.1074/jbc.M610028200 17114179

[B3] Alawin O. A. Ahmed R. A. Ibrahim B. A. Briski K. P. Sylvester P. W. (2016). Antiproliferative effects of γ-tocotrienol are associated with lipid raft disruption in HER2-positive human breast cancer cells. J. Nutr. Biochem. 27, 266–277. 10.1016/j.jnutbio.2015.09.018 26507543

[B4] Ali M. Y. Kim D. H. Seong S. H. Kim H.-R. Jung H. A. Choi J. S. (2017). α-Glucosidase and Protein Tyrosine Phosphatase 1B Inhibitory Activity of Plastoquinones from Marine Brown Alga Sargassum serratifolium. Marine Drugs 15(12). pii: E368. 10.3390/md15120368 29194348 PMC5742828

[B5] Alsabil K. Suor-Cherer S. Koeberle A. Viault G. Lavaud A. Temml,. V. (2016). Semisynthetic and Natural Garcinoic Acid Isoforms as New mPGES-1 Inhibitors. Planta Med. 82, 1110–1116. 10.1055/s-0042-108739 27286327

[B7] Azzi A. Boscoboinik D. Chatelain E. Ozer N. K. Stäuble B. (1993). d-alpha-tocopherol control of cell proliferation. Mol. Aspects Med. 14, 265–271. 10.1016/0098-2997(93)90014-5 8264342

[B6] Azzi A. (2019). Tocopherols, tocotrienols and tocomonoenols: Many similar molecules but only one vitamin E. Redox Biol. 26, 101259. 10.1016/j.redox.2019.101259 31254734 PMC6604160

[B8] Bartolini D. Franco F. D. Torquato P. Marinelli R. Cerra B. Ronchetti R. (2019). Garcinoic acid is a natural and selective agonist of Pregnane X Receptor. ChemRxiv. 10.1021/acs.jmedchem.0c00012 PMC7901650 32160459

[B9] Beharka A. A. Wu D. Serafini M. Meydani S. N. (2002). Mechanism of vitamin E inhibition of cyclooxygenase activity in macrophages from old mice: role of peroxynitrite. Free Radical Biol. Med. 32, 503–511. 10.1016/s0891-5849(01)00817-6 11958951

[B12] Birringer M. Pfluger P. Kluth D. Landes N. Brigelius-Flohé R. (2002). Identities and differences in the metabolism of tocotrienols and tocopherols in HepG2 cells. J. Nutr. 132, 3113–3118. 10.1093/jn/131.10.3113 12368403

[B10] Birringer M. EyTina J. H. Salvatore B. A. Neuzil J. (2003). Vitamin E analogues as inducers of apoptosis. Structure-function relation. Br. J. Cancer 88, 1948–1955. 10.1038/sj.bjc.6600981 12799642 PMC2741106

[B11] Birringer M. Lington D. Vertuani S. Manfredini S. Scharlau D. Glei M. (2010). Proapoptotic effects of long-chain vitamin E metabolites in HepG2 cells are mediated by oxidative stress. Free Radical Biol. Med. 49, 1315–1322. 10.1016/j.freeradbiomed.2010.07.024 20692332

[B13] Birringer M. Siems K. Maxones A. Frank J. Lorkowski S. (2018). Natural 6-hydroxy-chromanols and -chromenols. Structural diversity, biosynthetic pathways and health implications. RSC Adv. 8, 4803–4841. 10.1039/C7RA11819H PMC9078042 35539527

[B14] Brasky T. M. Felix A. S. Cohn D. E. McMeekin D. S. Mutch D. G. Creasman W. T. (2017). Nonsteroidal Anti-inflammatory Drugs and Endometrial Carcinoma Mortality and Recurrence. J. Natl. Cancer Ins. 109, 1–10. 10.1093/jnci/djw251 PMC5161320 28376204

[B15] Brigelius-Flohé R. ed. (2009). Vitamin E: the shrew waiting to be tamed. Free. Radic. Biol. Med. 46 (5), 543–554. 10.1016/j.freeradbiomed.2008.12.007 19133328

[B16] Campbell S. E. Stone W. L. Lee S. Whaley S. Yang H. Qui M. (2006). Comparative effects of RRR-alpha- and RRR-gamma-tocopherol on proliferation and apoptosis in human colon cancer cell lines. BMC Cancer 6, 13. 10.1186/1471-2407-6-13 16417629 PMC1379650

[B17] Choi B. W. Ryu G. Park S. H. Kim E. S. Shin J. Roh S. S. (2007). Anticholinesterase activity of plastoquinones from Sargassum sagamianum. Lead compounds for Alzheimer’s disease therapy. Phytother. Res. 21, 423–426. 10.1002/ptr.2090 17236179

[B18] Ciffolilli S. Wallert M. Bartolini D. Krauth V. Werz O. Piroddi M. (2015). Human serum determination and in vitro anti-inflammatory activity of the vitamin E metabolite α-(13′-hydroxy)-6-hydroxychroman. Free Radical Biol. Med. 89, 952–962. 10.1016/j.freeradbiomed.2015.08.019 26454076

[B19] Dhillon A. S. Hagan S. Rath O. Kolch W. (2007). MAP kinase signalling pathways in cancer. Oncogene 26, 3279–3290. 10.1038/sj.onc.1210421 17496922

[B20] Dieber-Rotheneder M. Puhl H. Waeg G. Striegl G. Esterbauer H. (1991). Effect of oral supplementation with D-alpha-tocopherol on the vitamin E content of human low density lipoproteins and resistance to oxidation. J. Lipid Res. 32, 1325–1332. 1770314

[B21] Evans H. M. Bishop K. S.(1922). On The Existence of a hitherto unrecognized dietary factor essential for reproduction. Sci. (N} York N.Y.) 56, 650–651. 10.1126/science.56.1458.650 17838496

[B22] Freiser H. Jiang Q. (2009). Gamma-tocotrienol and gamma-tocopherol are primarily metabolized to conjugated 2-(beta-carboxyethyl)-6-hydroxy-2,7,8-trimethylchroman and sulfated long-chain carboxychromanols in rats. J. Nutr. 139, 884–889. 10.3945/jn.108.103309 19297424 PMC2714389

[B23] Fujisawa A. Dunlap W. C. Yamamoto Y. (2010). Vitamin E protection in the biochemical adaptation of marine organisms to cold-water environments. Comp. Biochem. Physiol. Part B Biochem. Mol. Biol. 157, 145–158. 10.1016/j.cbpb.2010.04.011 20427025

[B24] Galli F. Lee R. Dunster C. Kelly F. J. (2002). Gas chromatography mass spectrometry analysis of carboxyethyl-hydroxychroman metabolites of α- and γ-tocopherol in human plasma. Free Radical Biol. Med. 32, 333–340. 10.1016/S0891-5849(01)00800-0 11841923

[B25] Galli F. Stabile A. M. Betti M. Conte C. Pistilli A. Rende M. (2004). The effect of alpha- and gamma-tocopherol and their carboxyethyl hydroxychroman metabolites on prostate cancer cell proliferation. Arch. Biochem. Biophys. 423, 97–102. 10.1016/j.abb.2003.11.014 14871472

[B26] Gilligan M. M. Gartung A. Sulciner M. L. Norris P. C. Sukhatme V. P. Bielenberg D. R. (2019). Aspirin-triggered proresolving mediators stimulate resolution incancer. Proc. Natl. Acad. Sci. U. S. A. 116, 6292–6297. 10.1073/pnas.1804000116 30862734 PMC6442621

[B27] Giusepponi D. Torquato P. Bartolini D. Piroddi M. Birringer M. Lorkowski S. (2017). Determination of tocopherols and their metabolites by liquid-chromatography coupled with tandem mass spectrometry in human plasma and serum. Talanta 170, 552–561. 10.1016/j.talanta.2017.04.030 28501210

[B28] Gkouveris I. Nikitakis N. G. (2017). Role of JNK signaling in oral cancer: A mini review. Tumour Biol. 39 (6), 1010428317711659. 10.1177/1010428317711659 28639904

[B29] Glauert H. P. (2007). “Vitamin E and NF-κB Activation: A Review: Vitamins and Hormones. 76, 135–153. 10.1016/S0083-6729(07)76006-5 17628174

[B30] Grammas P. Hamdheydari L. Benaksas E. J. Mou S. Pye Q. N. Wechter W. J. (2004). Anti-inflammatory effects of tocopherol metabolites. Biochem. Biophys. Res. Commun. 319, 1047–1052. 10.1016/j.bbrc.2004.05.082 15184087

[B31] Guthrie N. Gapor A. Chambers A. F. Carroll K. K. (1997). Inhibition of proliferation of estrogen receptor-negative MDA-MB-435 and -positive MCF-7 human breast cancer cells by palm oil tocotrienols and tamoxifen, alone and in combination. J. Nutr. 127, 544S–548S. 10.1093/jn/127.3.544S 9082043

[B32] Gwon W.-G. Joung E.-J. Kwon M.-S. Lim S.-J. Utsuki T. Kim H.-R. (2017). Sargachromenol protects against vascular inflammation by preventing TNF-α-induced monocyte adhesion to primary endothelial cells via inhibition of NF-κB activation. Int. Immunopharmacol. 42, 81–89. 10.1016/j.intimp.2016.11.014 27902962

[B33] Gysin R. Azzi A. Visarius T. (2002). Gamma-tocopherol inhibits human cancer cell cycle progression and cell proliferation by down-regulation of cyclins. FASEB J.: Off. Publ. Fed. Am. Soc. Exp. Biol. 16, 1952–1954. 10.1096/fj.02-0362fje 12368234

[B34] He L. Mo H. Hadisusilo S. Qureshi A. A. Elson C. E. (1997). Isoprenoids suppress the growth of murine B16 melanomas in vitro and in vivo. J. Nutr. 127, 668–674. 10.1093/jn/127.5.668 9164984

[B36] Heo S.-J. Kim K.-N. Yoon W.-J. Oh C. Choi Y.-U. Affan A. (2011). Chromene induces apoptosis via caspase-3 activation in human leukemia HL-60 cells. Food Chem. Toxicol. 49, 1998–2004. 10.1016/j.fct.2011.05.011 21600262

[B35] Heo S.-J. Jang J. Ye B.-R. Kim M.-S. Yoon W.-J. Oh C. (2014). Chromene suppresses the activation of inflammatory mediators in lipopolysaccharide-stimulated RAW 264.7 cells. Food Chem. Toxicol. 67, 169–175. 10.1016/j.fct.2014.02.023 24593990

[B37] Hosomi A. Arita M. Sato Y. Kiyose C. Ueda T. Igarashi O. (1997). Affinity for alpha-tocopherol transfer protein as a determinant of the biological activities of vitamin E analogs. FEBS Lett. 409, 105–108. 10.1016/s0014-5793(97)00499-7 9199513

[B38] Hur S. Lee H. Kim Y. Lee B.-H. Shin J. Kim T.-Y. (2008). Sargaquinoic acid and sargachromenol, extracts of Sargassum sagamianum, induce apoptosis in HaCaT cells and mice skin. Its potentiation of UVB-induced apoptosis. Eur. J. Pharmacol. 582, 1–11. 10.1016/j.ejphar.2007.12.025 18243174

[B39] Husain K. Francois R. A. Yamauchi T. Perez M. Sebti S. M. Malafa M. P. (2011). Vitamin E δ-tocotrienol augments the antitumor activity of gemcitabine and suppresses constitutive NF-κB activation in pancreatic cancer. Mol. Cancer Ther. 10, 2363–2372. 10.1158/1535-7163.MCT-11-0424 21971120 PMC3237822

[B40] Im Lee J. Seo Y.(2011). Chromanols from Sargassum siliquastrum and their antioxidant activity in HT 1080 cells. Chem. Pharm. Bull. 59, 757–761. 10.1248/cpb.59.757 21628914

[B41] Infante J. P. (1999). A function for the vitamin E metabolite α-tocopherol quinone as an essential enzyme cofactor for the mitochondrial fatty acid desaturases. FEBS Lett. 446, 1–5. 10.1016/S0014-5793(99)00170-2 10100602

[B42] Jang K. H. Lee B. H. Choi B. W. Lee H.-S. Shin J.(2005). Chromenes from the brown alga Sargassum siliquastrum. J. Natural Prod. 68, 716–723. 10.1021/np058003i 15921416

[B43] Jang Y. Park N.-Y. Rostgaard-Hansen A. L. Huang J. Jiang Q. (2016). Vitamin E metabolite 13′-carboxychromanols inhibit pro-inflammatory enzymes, induce apoptosis and autophagy in human cancer cells by modulating sphingolipids and suppress colon tumor development in mice. Free Radical Biol. Med. 95, 190–199. 10.1016/j.freeradbiomed.2016.03.018 27016075 PMC4867259

[B45] Jiang Q. Elson-Schwab I. Courtemanche C. Ames B. N.(2000). Gamma-tocopherol and its major metabolite, in contrast to alpha-tocopherol, inhibit cyclooxygenase activity in macrophages and epithelial cells. Proc. Natl. Acad. Sci. U. S. A. 97, 11494–11499. 10.1073/pnas.200357097 11005841 PMC17228

[B46] Jiang Q. Freiser H. Wood K. V. Yin X. (2007). Identification and quantitation of novel vitamin E metabolites, sulfated long-chain carboxychromanols, in human A549 cells and in rats. J. Lipid Res. 48, 1221–1230. 10.1194/jlr.D700001-JLR200 17299205 PMC2185712

[B49] Jiang Q. Yin X. Lill M. A. Danielson M. L. Freiser H. Huang J. (2008). Long-chain carboxychromanols, metabolites of vitamin E, are potent inhibitors of cyclooxygenases. Proc. Natl. Acad. Sci. U. S. A. 105, 20464–20469. 10.1073/pnas.0810962106 19074288 PMC2629323

[B50] Jiang Z. Yin X. Jiang Q. (2011). Natural forms of vitamin E and 13′-carboxychromanol, a long-chain vitamin E metabolite, inhibit leukotriene generation from stimulated neutrophils by blocking calcium influx and suppressing 5-lipoxygenase activity, respectively. J. Immunol. (Baltimore Md. : 1950) 186, 1173–1179. 10.4049/jimmunol.1002342 PMC4050064 21169551

[B48] Jiang Q. Rao X. Kim C. Y. Freiser H. Zhang Q. Jiang,. Z. (2012). Gamma-tocotrienol induces apoptosis and autophagy in prostate cancer cells by increasing intracellular dihydrosphingosine and dihydroceramide. Int. J. Cancer 130, 685–693. 10.1002/ijc.26054 21400505 PMC3381336

[B47] Jiang Q. Jiang Z. Hall Y. J. Jang Y. Snyder P. W. Bain C. (2013). Gamma-tocopherol attenuates moderate but not severe colitis and suppresses moderate colitis-promoted colon tumorigenesis in mice. Free Radical Biol. Med. 65, 1069–1077. 10.1016/j.freeradbiomed.2013.08.187 24013093 PMC3859799

[B44] Jiang Q. (2014). Natural forms of vitamin E: metabolism, antioxidant, and anti-inflammatory activities and their role in disease prevention and therapy. Free Radical Biol. Med. 72, 76–90. 10.1016/j.freeradbiomed.2014.03.035 24704972 PMC4120831

[B51] Jiménez-Martínez M. Stamatakis K. Fresno M. (2019). The Dual-Specificity Phosphatase 10 (DUSP10): Its Role in Cancer, Inflammation, and Immunity. Int. J. Mol. Sci. 20 (7). pii: 1626. 10.3390/ijms20071626 PMC6480380 30939861

[B52] Johnson C. H. Slanař O. Krausz K. W. Kang D. W. Patterson A. D. Kim J.-H. (2012). Novel metabolites and roles for α-tocopherol in humans and mice discovered by mass spectrometry-based metabolomics. Am. J. Clin. Nutr. 96, 818–830. 10.3945/ajcn.112.042929 22952181 PMC3441109

[B53] Jurjus A. Eid A. Al Kattar S. Zeenny M. N. Gerges-Geagea A. Haydar H. (2016). Inflammatory bowel disease, colorectal cancer and type 2 diabetes mellitus: The links. BBA Clin. 5, 16–24. 10.1016/j.bbacli.2015.11.002 27051585 PMC4802401

[B54] Kaminska B. (2005). MAPK signalling pathways as molecular targets for anti-inflammatory therapy–from molecular mechanisms to therapeutic benefits. Biochim. Biophys. Acta 1754, 253–262. 10.1016/j.bbapap.2005.08.017 16198162

[B56] Kim J.-A. Ahn B.-N. Kong C.-S. Kim S.-K. (2012). Protective effect of chromene isolated from Sargassum horneri against UV-A-induced damage in skin dermal fibroblasts. Exp. Dermatol. 21, 630–631. 10.1111/j.1600-0625.2012.01535.x 22775999

[B57] Kim S. Lee M.-S. Lee B. Gwon W.-G. Joung E.-J. Yoon N.-Y. (2014). Anti-inflammatory effects of sargachromenol-rich ethanolic extract of Myagropsis myagroides on lipopolysaccharide-stimulated BV-2 cells. BMC Complementary Altern. Med. 14, 231. 10.1186/1472-6882-14-231 PMC4227293 25005778

[B59] Kim Y. Wang W. Okla M. Kang I. Moreau R. Chung S. (2016). Suppression of NLRP3 inflammasome by γ-tocotrienol ameliorates type 2 diabetes. J. Lipid Res. 57, 66–76. 10.1194/jlr.M062828 26628639 PMC4689338

[B58] Kim Y. Gromovsky A. D. Brown J. M. Chung S. (2018). Gamma-tocotrienol attenuates the aberrant lipid mediator production in NLRP3 inflammasome-stimulated macrophages. J. Nutr. Biochem. 58, 169–177. 10.1016/j.jnutbio.2018.05.007 29957361 PMC6095813

[B55] Kim H. K.(2014). Role of ERK/MAPK signalling pathway in anti-inflammatory effects of Ecklonia cava in activated human mast cell line-1 cells. Asian Pac. J. Trop. Med. 7, 703–708. 10.1016/S1995-7645(14)60120-6

[B60] Klein E. A. Thompson I. M. Tangen C. M. Crowley J. J. Lucia M. S. Goodman P. J. (2011). Vitamin E and the risk of prostate cancer: the Selenium and Vitamin E Cancer Prevention Trial (SELECT). JAMA 306, 1549–1556. 10.1001/jama.2011.1437 21990298 PMC4169010

[B61] Kluge S. Schubert M. Schmölz L. Birringer M. Wallert M. Lorkowski S. (2016). “Garcinoic Acid,” in Studies in natural products chemistry, vol. Volume 51 . Ed. Rahman A.-u. (Amsterdam, Netherlands: Elsevier), 435–481.

[B62] Kruk J. Pisarski A. Szymańska R. (2011). Novel vitamin E forms in leaves of Kalanchoe daigremontiana and Phaseolus coccineus. J. Plant Physiol. 168, 2021–2027. 10.1016/j.jplph.2011.06.015 21856038

[B64] Kunnumakkara A. B. Sung B. Ravindran J. Diagaradjane P. Deorukhkar A. Dey S. (2010). {Gamma}-tocotrienol inhibits pancreatic tumors and sensitizes them to gemcitabine treatment by modulating the inflammatory microenvironment. Cancer Res. 70, 8695–8705. 10.1158/0008-5472.CAN-10-2318 20864511 PMC2970705

[B63] Kunnumakkara A. B. Sailo B. L. Banik K. Harsha C. Prasad S. Gupta S. C. (2018). Chronic diseases, inflammation, and spices: how are they linked? J. Trans. Med. 16, 14. 10.1186/s12967-018-1381-2 PMC5785894 29370858

[B65] Kusumi T. Shibata Y. Ishitsuka M. Kinoshita T. Kakisawa H. (1979). Structures of new Plastoquinones from the brown alga sargassum serratifolium. Chem. Lett. 8, 277–278. 10.1246/cl.1979.277

[B66] Lasry A. Ben-Neriah Y. (2015). Senescence-associated inflammatory responses: aging and cancer perspectives. Trends Immunol. 36, 217–228. 10.1016/j.it.2015.02.009 25801910

[B68] Lavaud A. Richomme P. Litaudon M. Andriantsitohaina R. Guilet D.(2013). Antiangiogenic tocotrienol derivatives from Garcinia amplexicaulis. J. Natural Prod. 76, 2246–2252. 10.1021/np400598y 24245984

[B67] Lavaud A. Richomme P. Gatto J. Aumond M.-C. Poullain C. Litaudon M. (2015). A tocotrienol series with an oxidative terminal prenyl unit from Garcinia amplexicaulis. Phytochemistry 109, 103–110. 10.1016/j.phytochem.2014.10.024 25468538

[B69] Lee J.-H. Ko J.-Y. Samarakoon K. Oh J.-Y. Heo S.-J. Kim C.-Y. (2013). Preparative isolation of sargachromanol E from Sargassum siliquastrum by centrifugal partition chromatography and its anti-inflammatory activity. Food Chem. Toxicol. 62, 54–60. 10.1016/j.fct.2013.08.010 23973192

[B70] Liebler D. C. Baker P. F. Kaysen K. L. (1990). Oxidation of vitamin E: evidence for competing autoxidation and peroxyl radical trapping reactions of the tocopheroxyl radical. J. Am. Chem. Soc. 112, 6995–7000. 10.1021/ja00175a037

[B71] Lim S. Choi A.-H. Kwon M. Joung E.-J. Shin T. Lee S.-G. (2019). Evaluation of antioxidant activities of various solvent extract from Sargassum serratifolium and its major antioxidant components. Food Chem. 278, 178–184. 10.1016/j.foodchem.2018.11.058 30583359

[B72] Lippman S. M. Klein E. A. Goodman P. J. Lucia M. S. Thompson I. M. Ford L. G. (2009). Effect of selenium and vitamin E on risk of prostate cancer and other cancers: the Selenium and Vitamin E Cancer Prevention Trial (SELECT). JAMA 301, 39–51. 10.1001/jama.2008.864 19066370 PMC3682779

[B73] Loganathan R. Selvaduray K. R. Nesaretnam K. Radhakrishnan A. K. (2013). Tocotrienols promote apoptosis in human breast cancer cells by inducing poly(ADP-ribose) polymerase cleavage and inhibiting nuclear factor kappa-B activity. Cell Proliferation 46, 203–213. 10.1111/cpr.12014 23510475 PMC6603791

[B74] Maloney D. J. Hecht S. M. (2005). A stereocontrolled synthesis of delta-trans-tocotrienoloic acid. Organic Lett. 7, 4297–4300. 10.1021/ol051849t 16146411

[B75] Mazzini F. Betti M. Netscher T. Galli F. Salvadori P. (2009). Configuration of the vitamin E analogue garcinoic acid extracted from Garcinia Kola seeds. Chirality 21, 519–524. 10.1002/chir.20630 18655162

[B76] Merchant N. Bhaskar L. V. K. S. Momin S. Sujatha P. Reddy A. B. M. Nagaraju G. P. (2018). 5-Lipoxygenase: Its involvement in gastrointestinal malignancies. Crit. Rev. Oncology/Hematology 127, 50–55. 10.1016/j.critrevonc.2018.05.012 29891111

[B77] Meydani S. N. Barklund M. P. Liu S. Meydani M. Miller R. A. Cannon J. G. (1990). Vitamin E supplementation enhances cell-mediated immunity in healthy elderly subjects. Am. J. Clin. Nutr. 52, 557–563. 10.1093/ajcn/52.3.557 2203257

[B78] Moossavi M. Parsamanesh N. Bahrami A. Atkin S. L. Sahebkar A. (2018). Role of the NLRP3 inflammasome in cancer. Mol. Cancer 17, 158. 10.1186/s12943-018-0900-3 30447690 PMC6240225

[B79] Nesaretnam K. Guthrie N. Chambers A. F. Carroll K. K. (1995). Effect of tocotrienols on the growth of a human breast cancer cell line in culture. Lipids 30, 1139–1143. 10.1007/bf02536615 8614304

[B80] Nesaretnam K. Stephen R. Dils R. Darbre P. (1998). Tocotrienols inhibit the growth of human breast cancer cells irrespective of estrogen receptor status. Lipids 33, 461–469. 10.1007/s11745-998-0229-3 9625593

[B81] Pak W.-M. Kim K.-B.-W. R. Kim M.-J. Cho J.-Y. Ahn D.-H. (2015). Inhibitory effect of hexane fraction from Myagropsis myagroides on pancreatic α-amylase in vitro. J. Microbiol. Biotechnol. 25, 328–333. 10.4014/jmb.1409.09012 25315050

[B82] Parker R. S. Sontag T. J. Swanson J. E. (2000). Cytochrome P4503A-dependent metabolism of tocopherols and inhibition by sesamin. Biochem. Biophys. Res. Commun. 277, 531–534. 10.1006/bbrc.2000.3706 11061988

[B83] Pein H. Ville A. Pace S. Temml V. Garscha U. Raasch,. M. (2018). Endogenous metabolites of vitamin E limit inflammation by targeting 5-lipoxygenase. Nat. Commun. 9, 3834. 10.1038/s41467-018-06158-5 30237488 PMC6148290

[B84] Péter S. Friedel A. Roos F. F. Wyss A. Eggersdorfer M. Hoffmann,. K. (2015). A Systematic Review of Global Alpha-Tocopherol Status as Assessed by Nutritional Intake Levels and Blood Serum Concentrations. Int. J. Vitamin Nutr. Res. 85, 261–281. 10.1024/0300-9831/a000281 27414419

[B85] Podszun M. C. Jakobi M. Birringer M. Weiss J. Frank J. (2017). The long chain α-tocopherol metabolite α-13′-COOH and γ-tocotrienol induce P-glycoprotein expression and activity by activation of the pregnane X receptor in the intestinal cell line LS 180. Mol. Nutr. Food Res. 61 (3). 10.1002/mnfr.201600605 27714977

[B86] Rådmark O. Werz O. Steinhilber D. Samuelsson B. (2015). 5-Lipoxygenase, a key enzyme for leukotriene biosynthesis in health and disease. Biochim. Biophys. Acta (BBA) - Mol. Cell Biol. Lipids 1851, 331–339. 10.1016/j.bbalip.2014.08.012 25152163

[B87] Reddanna P. Krishna Rao M. Channa Reddy C. (1985). Inhibition of 5-lipoxygenase by vitamin E. FEBS Lett. 193, 39–43. 10.1016/0014-5793(85)80075-2 3934003

[B88] Richomme P. Helesbeux J.-J. Guilet D. Seraphin D. Stuppner H. Waltenberger B. (2017) 2, 2017. WO2017032881A1, filed March.

[B89] Rimbach G. Moehring J. Huebbe P. Lodge J. K. (2010). Gene-regulatory activity of alpha-tocopherol. Mol. (Basel Switzerland) 15, 1746–1761. 10.3390/molecules15031746 PMC6257188 20336011

[B90] Roos J. Grösch S. Werz O. Schröder P. Ziegler S. Fulda S. (2016). Regulation of tumorigenic Wnt signaling by cyclooxygenase-2, 5-lipoxygenase and their pharmacological inhibitors: A basis for novel drugs targeting cancer cells? Pharmacol. Ther. 157, 43–64. 10.1016/j.pharmthera.2015.11.001 26549540

[B91] Sakai M. Okabe M. Yamasaki M. Tachibana H. Yamada K. (2004). Induction of apoptosis by tocotrienol in rat hepatoma dRLh-84 cells. Anticancer Res. 24, 1683–1688. 15274341

[B92] Schmölz L. Birringer M. Lorkowski S. Wallert M. (2016). Complexity of vitamin E metabolism. World J. Biol. Chem. 7, 14–43. 10.4331/wjbc.v7.i1.14 26981194 PMC4768118

[B93] Schmölz L. Wallert M. Rozzino N. Cignarella A. Galli F. Glei,. M. (2017). Structure-function relationship studies in vitro reveal distinct and specific effects of long-chain metabolites of Vitamin E. Mol. Nutr. Food Res. 61(12). 10.1002/mnfr.201700562 28960703

[B94] Serhan C. N. (2014). Pro-resolving lipid mediators are leads for resolution physiology. Nature 510, 92–101. 10.1038/nature13479 24899309 PMC4263681

[B95] Silva D. H. S. Zhang Y. Santos L. A. Bolzani V. S. Nair M. G. (2007). Lipoperoxidation and cyclooxygenases 1 and 2 inhibitory compounds from Iryanthera juruensis. J. Agric. Food Chem. 55, 2569–2574. 10.1021/jf063451x 17335225

[B96] Sontag T. J. Parker R. S. (2002). Cytochrome P450 omega-hydroxylase pathway of tocopherol catabolism. Novel mechanism of regulation of vitamin E status. J. Biol. Chem. 277, 25290–25296. 10.1074/jbc.M201466200 11997390

[B97] Swanson K. V. Deng M. Ting J. P.-Y. (2019). The NLRP3 inflammasome: molecular activation and regulation to therapeutics. Nat. Rev. Immunol. 19, 477–489. 10.1038/s41577-019-0165-0 31036962 PMC7807242

[B98] Tan P. H. Sagoo P. Chan C. Yates J. B. Campbell J. Beutelspacher S. C. (2005). Inhibition of NF-kappa B and oxidative pathways in human dendritic cells by antioxidative vitamins generates regulatory T cells. J. Immunol. (Baltimore Md. : 1950) 174, 7633–7644. 10.4049/jimmunol.174.12.7633 15944264

[B99] Tsang C. K. Ina A. Goto T. Kamei Y. (2005). Sargachromenol, a novel nerve growth factor-potentiating substance isolated from Sargassum macrocarpum, promotes neurite outgrowth and survival via distinct signaling pathways in PC12D cells. Neuroscience 132, 633–643. 10.1016/j.neuroscience.2005.01.028 15837125

[B100] Virtamo J. Taylor P. R. Kontto J. Männistö S. Utriainen M. Weinstein S. J. (2014). Effects of α-tocopherol and β-carotene supplementation on cancer incidence and mortality: 18-year postintervention follow-up of the Alpha-tocopherol, Beta-carotene Cancer Prevention Study. Int. J. Cancer 135, 178–185. 10.1002/ijc.28641 24338499 PMC3991754

[B102] Wallert M. Mosig S. Rennert K. Funke H. Ristow M. Pellegrino R. M. (2014a). Long-chain metabolites of α-tocopherol occur in human serum and inhibit macrophage foam cell formation in vitro. Free Radical Biol. Med. 68, 43–51. 10.1016/j.freeradbiomed.2013.11.009 24296243

[B103] Wallert M. Schmölz L. Galli F. Birringer M. Lorkowski S. (Eds.) (2014b). Regulatory metabolites of vitamin E and their putative relevance for atherogenesis, Redox Biol. 2, 495–503. 10.1016/j.redox.2014.02.002 24624339 PMC3949092

[B104] Wallert M. Schmölz L. Koeberle A. Krauth V. Glei M. Galli,. F. (2015). α-Tocopherol long-chain metabolite α-13′-COOH affects the inflammatory response of lipopolysaccharide-activated murine RAW264.7 macrophages. Mol. Nutr. Food Res. 59, 1524–1534. 10.1002/mnfr.201400737 25943249

[B101] Wallert M. Bauer J. Kluge S. Schmölz L. Chen Y.-C. Ziegler M. (2019). The vitamin E derivative garcinoic acid from Garcinia kola nut seeds attenuates the inflammatory response. Redox Biol. 24, 101166. 10.1016/j.redox.2019.101166 30897408 PMC6426704

[B105] Wang Y. Park N.-Y. Jang Y. Ma A. Jiang Q. (2015). Vitamin E γ-tocotrienol inhibits cytokine-stimulated NF-κB activation by induction of anti-inflammatory A20 via stress adaptive response due to modulation of Sphingolipids. J. Immunol. (Baltimore Md. : 1950) 195, 126–133. 10.4049/jimmunol.1403149 PMC4475472 26002975

[B106] Wculek S. K. Malanchi I. (2015). Neutrophils support lung colonization of metastasis-initiating breast cancer cells. Nature 528, 413–417. 10.1038/nature16140 26649828 PMC4700594

[B107] Yam M.-L. Abdul Hafid S. R. Cheng H.-M. Nesaretnam K. (2009). Tocotrienols suppress proinflammatory markers and cyclooxygenase-2 expression in RAW264.7 macrophages. Lipids 44, 787–797. 10.1007/s11745-009-3326-2 19655189

[B108] Yang E.-J. Ham Y. M. Yang K.-W. Lee N. H. Hyun C.-G. (2013). Sargachromenol from Sargassum micracanthum inhibits the lipopolysaccharide-induced production of inflammatory mediators in RAW 264.7 macrophages. Sci. World J. 2013, 712303. 10.1155/2013/712303 PMC3806450 24194688

[B109] Yap W. N. Chang P. N. Han H. Y. Lee D. T. W. Ling M. T. Wong Y. C. (2008). Gamma-tocotrienol suppresses prostate cancer cell proliferation and invasion through multiple-signalling pathways. Br. J. Cancer 99, 1832–1841. 10.1038/sj.bjc.6604763 19002171 PMC2600692

[B110] Yoon W.-J. Heo S.-J. Han S.-C. Lee H.-J. Kang G.-J. Kang H.-K. (2012a). Anti-inflammatory effect of sargachromanol G isolated from Sargassum siliquastrum in RAW 264.7 cells. Arch. Pharmacal Res. 35, 1421–1430. 10.1007/s12272-012-0812-5 22941485

[B111] Yoon W.-J. Heo S.-J. Han S.-C. Lee H.-J. Kang G.-J. Yang E.-J. (2012b). Sargachromanol G regulates the expression of osteoclastogenic factors in human osteoblast-like MG-63 cells. Food Chem. Toxicol. 50, 3273–3279. 10.1016/j.fct.2012.06.022 22727857

[B112] Yoon W.-J. Kim K.-N. Heo S.-J. Han S.-C. Kim J. Ko Y.-J. (2013). Sargachromanol G inhibits osteoclastogenesis by suppressing the activation NF-κB and MAPKs in RANKL-induced RAW 264.7 cells. Biochem. Biophys. Res. Commun. 434, 892–897. 10.1016/j.bbrc.2013.04.046 23611776

[B113] Zhao Y. Lee M.-J. Cheung C. Ju J.-H. Chen Y.-K. Liu B. (2010). Analysis of multiple metabolites of tocopherols and tocotrienols in mice and humans. J. Agric. Food Chem. 58, 4844–4852. 10.1021/jf904464u 20222730 PMC2858244

[B114] Zingg J.-M. (2019). Vitamin E: Regulatory Role on Signal Transduction. IUBMB Life 71, 456–478. 10.1002/iub.1986 30556637

